# Recent Advances on Application of Modified Biochar for the Removal of Pharmaceutical Compounds from Wastewater

**DOI:** 10.1002/open.70221

**Published:** 2026-05-22

**Authors:** Ebrahim Tangestani, Ravinder Kumar, Catherine M. Miller, Elsa Antunes

**Affiliations:** ^1^ College of Science and Engineering James Cook University Townsville Queensland Australia; ^2^ College of Medicine and Dentistry James Cook University Cairns Queensland Australia

**Keywords:** adsorption mechanisms, biochar modification, biochar regeneration, biochar sustainability, pharmaceutical compounds

## Abstract

The widespread use of pharmaceutical compounds (PCs) has led to their extensive contamination in aquatic environments. Biochar, a carbon‐rich and porous material, offers a promising solution for the remediation of PCs, particularly when modified. Modification methods can be physical (ball milling, steam activation, and CO_2_ activation) or chemical (acid/alkali treatment, oxidation, surface functionalization, metal impregnation, and nano structuring). These treatments enhance biochar's structural properties including pore size, pore volume, surface area, and surface functionality, thereby increasing its affinity for PCs and improving removal efficiency. Modified biochar enables multiple adsorption mechanisms, including pore diffusion, electrostatic interactions, hydrophobic effects, hydrogen bonding, and π–π interactions, which act synergistically to remove PCs from water. This review critically discusses various biochar modification techniques, their efficacies, and key parameters influencing their performance. It also highlights adsorption mechanisms, regeneration strategies, and the importance of biochar stability for economic and environmental feasibility. Additionally, life cycle and techno‐economic analyses are discussed to evaluate the financial and technical viability of using modified biochar for PCs' remediation. Finally, the review outlines the major challenges associated with modification techniques and provides insights into future research directions to enhance the removal of PCs from aqueous environments.

## Graphical abstract

Modified biochar is an effective adsorbent for removing pharmaceutical contaminants from water. Physical and chemical modifications enhance its surface area, porosity, and functionality, enabling multiple adsorption mechanisms. These synergistic interactions improve removal efficiency. This review evaluates modification methods, mechanisms, regeneration, and techno‐economic feasibility, while highlighting challenges and future directions for sustainable water remediation.

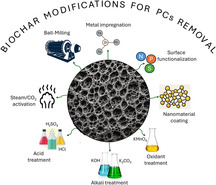

## 1 Introduction

**FIGURE 1:**
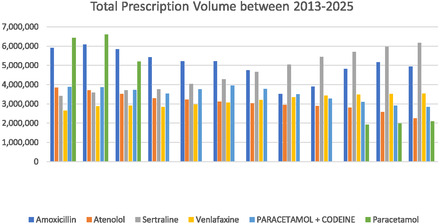
The total prescription volume of some pharmaceuticals in Australia “between” 2013 and 2025 according to Australia, Pharmaceutical Benefits Scheme (PBS) Expenditure [5, 6].

Pharmaceutical compounds (PCs), such as antibiotics, hormones, and antidepressants, have seen increased consumption in recent decades due to population growth, advancements in healthcare, research, global availability, and aging populations in industrialized nations [1]. For instance, global antibiotic consumption enhanced by 65% from 2000 to 2015 [2], and it is predicted that the use of antibacterial drugs will increase by another 200% by 2030 [2]. Additionally, over the period of the COVID‐19 pandemic, there was an unparalleled surge in the employment of drugs and medications, particularly antidepressants, resulting from the widespread depressive and anxious states experienced by the individuals [3, 4]. According to the Pharmaceutical Benefits Scheme Expenditure & Prescriptions report, the prescription volume of venlafaxine in Australia increased from 2,870,523 to 3,532,495 between 2014 and 30 June 2025 [5, 6]. Total prescription volume of some PCs in Australia can be seen in Figure 1. These volumes underscore the importance of assessing the potential environmental pollution caused by these medications.

The increase in consumption of PCs worldwide has also created a new challenge of their contamination in various waste streams like groundwater, aquatic surface resources, wastewater, lakes, rivers, and seawater, with concentrations varying between a few ng/L to hundreds of µg/L [7]. These PCs can persist in water sources and have the potential to bioaccumulate in organisms [8], potentially leading to endocrine disorders [9] and drug resistance [10]. Elevated levels of these compounds can potentially impact aquatic ecosystems, wildlife, and contribute to chronic diseases [11, 12, 13, 14]; thus, these chemicals represents a serious risk to both environmental preservation and the human well‐being [12, 15, 16]. For example, environmental contamination by PCs has been associated with the rise of antimicrobial resistance in microorganisms, a pressing global public health issue recognized by the United Nations [17]. Addressing the environmental presence of PCs and their role in antimicrobial resistance is key to preserve the effectiveness of pharmaceuticals and promote public health safety [18, 19].

Modern wastewater treatment plants (WWTPs) use filtration methods using membranes, including ultrafiltration (UF), nanofiltration (NF), reverse osmosis (RO), and microfiltration (MF) to remove pharmaceutical pollutants. These membranes effectively remove suspended solids but are less efficient at eliminating PCs due to their molecular weights [20]. These technologies also face other challenges, including membrane fouling [21], low permeability, high operational costs, and high energy demand since NF/RO requires high energy input to overcome osmotic pressure and drive water through the membrane [22, 23]. Alternatively, the adsorption method has been proven as a capable and versatile approach for removing PCs from wastewater because it can capture trace‐level contaminants with high efficiency [24]. In this context, the development of advanced adsorbent materials has gained increasing attention as an effective strategy to overcome the limitations of conventional treatment technologies and improve the removal efficiency of emerging contaminants from water systems [25]. The adsorption technique employs high surface area, and chemically tailored adsorbents such as activated carbon (AC) or modified biochar allows strong binding through mechanisms like hydrophobic, electrostatic attraction, and π–π stacking [26]. One investigation demonstrated that biochar from pyrolysis of pine chips achieved treatment efficiencies of 94.1% and 97.7% for acetaminophen (ACM) and naproxen, respectively, outperforming commercial AC, which had removal rates of 81.6% and 94.1% [27]. Furthermore, biochar offers significant advantages over other advanced materials such as AC, carbon nanotubes, and graphene, which, despite their high performance, are often associated with high production costs and complex synthesis processes. In contrast, biochar is a more sustainable and cost‐effective alternative. Compared to microorganisms and fungi, which may suffer from stability limitations, and zeolites, biochar provides an optimal balance of low cost, wide availability, tunable surface properties, and broad adsorption capability. Therefore, biochar represents a promising and scalable adsorbent for water and wastewater treatment applications [28].

Though biochar exhibits significant potential for PCs' removal from wastewater, its efficacy may be enhanced even more by altering its physical and chemical characteristics through various approaches, including physical and chemical modifications. Physical modification of biochar may involve typical methods like ball‐milling, steam activation, and CO_2_ activation, which enhances its structural features such as particle size, pore size, and surface area [29, 30]. Alternatively, chemical modification of biochar enhances its physical texture and chemical functionality characteristics, particularly its surface functionality, reactivity, and selectivity toward specific PCs and can be categorized into various types like acid treatment, alkali treatment, oxidant treatment, surface functionalization, metal impregnation, and nanostructured biochar [31, 32, 33]. For example, treating biochar with acids such as HCl or H_2_SO_4_ generally introduces functional groups containing oxygen, namely hydroxyl and carboxyl. These functional groups can strengthen electrostatic interactions and hydrogen bonding with polar PCs, thereby enhancing their removal. Research showcased the practical application of biochar modified with 2.5 M H_2_SO_4_ and evaluated for its ability to remove carbamazepine and ibuprofen [24]. The H_2_SO_4_‐treated biochar demonstrated approximately a 75% increase in removal efficiency. Peak adsorption performance was reported as 51.7 mg/g for carbamazepine and 38.8 mg/g for ibuprofen. The proposed adsorption mechanisms included diffusion through channel π–π interactions along with van der Waals interactions and hydrogen bonding [24]. Similarly, other modification methods have shown promising results for PC removal. A study by Wang et al. [34] developed a boron‐doped biochar, followed by peroxydisulfate activation for the adsorption of tetracycline (TC). The boron‐doping process introduced functional groups like BCO_2_ and BC_2_O on the surface of the biochar [34]. As a result, the modified biochar exhibited remarkably enhanced adsorption performance for TC, achieving 90% removal within 30 min compared to the undoped version [34].

Biochar modification presents a promising strategy for engineering advanced biochar materials with enhanced functionality for the mitigation of PCs. Throughout the past decade, numerous studies have explored various modification techniques to improve biochar's performance in this context. Therefore, some review articles have been published in last 5 years on this subject. For example, Kang et al. [35] published a review article on the removal of pharmaceuticals using pristine biochar via adsorption and persulfate‐based advanced oxidation processes (AOPs), with emphasis on degradation pathways and attack sites. Another review article published by Chauhan et al. [36] provides a general overview of biochar‐based adsorption for pharmaceutical removal, highlighting advantages, knowledge gaps, and future research needs. Recently, Nand et al. [37] overviewed adsorption efficiencies of pristine biochar for various pharmaceuticals and discussed adsorption mechanisms, while more recently Munuhe et al. [38], reviewed various adsorbents (biochar, nanomaterials, and composites) for PCs removal, with emphasis on adsorption capacities and emerging nanocomposites. However, none of these articles discussed biochar modification methods on PCs' removal. Compared with previously published reviews, which primarily focus on pristine biochar, general adsorption performance, or a broad range of adsorbents, this review offers a distinct and novel contribution by systematically centering on modified biochar for the removal of PCs from aqueous environments. Unlike earlier studies that only briefly mention modification or discuss adsorption mechanisms in a generic manner, this review comprehensively classifies physical, chemical, and nanostructured biochar modification strategies and explicitly links these modifications to changes in structural properties, surface functionality, and dominant adsorption mechanisms. Furthermore, it uniquely addresses biochar regeneration and stability, which are critical for long‐term application but largely overlooked in previous reviews. Most importantly, this is the first review to integrate life cycle assessment (LCA) and techno‐economic analysis to evaluate the environmental sustainability, financial viability, and technical feasibility of modified biochar systems, thereby bridging the gap between laboratory‐scale research and practical implementation and providing a forward‐looking framework for future development of biochar‐based pharmaceutical remediation technologies.

## 2 Methodology

A total of 124 published articles were thoroughly reviewed and added in this study. Relevant literature was retrieved from multiple databases, including Scopus, ScienceDirect, and Google Scholar, using keywords such as *modified biochar*, *modified biochar for pharmaceutical removal*, *biochar treatment*, *pharmaceutical removal from wastewater*, and *life cycle assessment of modified biochar application*. For specific sections, some keywords like *alkali treatment of biochar*, *acid treatment of biochar*, *surface functionalization of biochar* were also used to acquire the relevant literature.

Nearly 200 articles were reviewed by the authors, however, priority was given to studies published within the last 5–6 years to ensure the inclusion of the most recent and relevant findings. Consequently, 97 of the selected articles were published between 2020 and 2025, while the remaining 27 articles dated from 2007 to 2019. This indicates that the article delivers current, cutting‐edge insights into biochar modification strategies for the effective removal of PCs.

### 2.1 PCs in Wastewater

PCs can enter the environmental systems through diverse sources and routes, including wastewater effluent, improper disposal, landfills, agricultural runoff, aquaculture, and manufacturing [7, 39, 40]. Once PCs are released into the environment via wastewater or agricultural runoff, processes like absorption, distribution, metabolism, and excretion govern their fate. As a result of their high bioavailability and resistance to degradation, PCs can persist in water, soil, and wildlife, leading to unintended ecosystem exposure, harming aquatic life, and contributing to antimicrobial resistance. Recognizing these processes is vital for assessing environmental threats and devising practical reduction and regulatory strategies [41, 42, 43]. For instance, when zebrafish were exposed to low concentrations of TC, their swimming patterns changed due to TC‐induced photosensitivity. These effects were partially reversed once exposure ceased [44]. Similarly, Yaqui‐Tang et al. [45] reported that zebrafish exposed to venlafaxine at concentrations of 1, 10, and 100 μg/L for 20 days exhibited disrupted courtship behavior, attributed to alterations in brain neurotransmitter levels [45]. Other studies have also shown that sertraline negatively affects zebrafish reproduction, boldness, anxiety, and sociability [46, 47]. Table 1 and Tables S1–S4 outlines major PCs detected in different aqueous streams in Australia and other countries in the globe, which indicate widespread distribution of pharmaceutical pollutants in aqueous streams. This observation is well aligned with the prescription trends presented in Figure 1, where pharmaceuticals with higher consumption rates tend to correspond to higher detection frequencies and concentrations in environmental samples. Particularly, the concentrations of antidepressants like sertraline and venlafaxine, and antibiotics like amoxicillin (AMX) have been found significantly high in both the effluent and influent of wastewater treatment facilities. Unfortunately, conventional wastewater treatments like biological processes, coagulation/flocculation, sedimentation, and filtration are ineffective in removing PCs from water [53, 54]. Therefore, more advanced systems are required to remove PCs even at low‐level concentrations. In this regard, the adsorption process could be a highly feasible and advantageous alternative. Moreover, utilization of sustainable materials like biochar can make the process more environmentally friendly. In further sections, biochar utilization and its modified forms for controlling PCs removal have been discussed in detail.

**TABLE 1:** PCs residues detected in the Australian environment (according to The National Toxics Network (NTN) in 2015 and EPA Victoria's report in 2023) and prescription volume in Australia (according to Pharmaceutical Benefits Scheme (PBS) prescriptions & expenditure summary: 1 July 2022 to 30 June 2023).

Pharmaceutical	Therapeutic use	Source	Location	Average concentration, ng/L	Maximum concentration, ng/L	Detection Method
Amoxicillin	Antibiotic	WWTP effluent River waters	Queensland [48] Queensland [48]	—	50 200	LC–MS/MS [49] LC–MS/MS [49]
Atenolol	Beta blocker	Various river systems Influent (raw sewage) Effluent (treated wastewater)	Around Australia [48] Recycled water in Victoria [50] Recycled water in Victoria [50]	2120 80	133 4900 µg/L 560	LC–MS/MS [51] LC–MS/MS [50] LC–MS/MS [50]
Ciprofloxacin	Antibiotic	WWTP effluent River waters	Brisbane [48] Queensland [48]	640	742 1300	LC–MS/MS [52] LC–MS/MS [49]
Paracetamol	Analgesic	Various river systems Influent (raw sewage) Effluent (treated wastewater)	Around Australia [48] Recycled water in Victoria [50] Recycled water in Victoria [50]	85 247 200 40	7150 470 000 720	LC–MS/MS [51] LC–MS/MS [50] LC–MS/MS [50]
Tetracycline	Antibiotic	WWTP effluent River waters	Queensland [48] Queensland [48]	20 80	—	LC–MS/MS [49] LC–MS/MS [49]
Trimethoprim	Antibiotic	WWTP effluent WWTP effluent River waters Various river system Influent (raw sewage) Effluent (treated wastewater)	Brisbane [48] Queensland [48] Queensland [48] Around Australia [48] Recycled water in Victoria [50] Recycled water in Victoria [50]	50 10 3 240 50	70 250 150 657 30 000 340	LC–MS/MS [52] LC–MS/MS [49] LC–MS/MS [49] LC–MS/MS [51] LC–MS/MS [50] LC–MS/MS [50]
Sulfamethoxazole	Antibiotic	WWTP effluent WWTP effluent River waters Various river systems Influent (raw sewage) Effluent (treated wastewater)	Brisbane [48] Queensland [48] Queensland [48] Around Australia [48] Victoria's Recycled Water [50] Victoria's Recycled Water [50]	270 50 8 740 110	320 200 2000 67 1500 650	LC–MS/MS [52] LC–MS/MS [49] LC–MS/MS [49] LC–MS/MS [51] LC–MS/MS [50] LC–MS/MS [50]
Sertraline	Antidepressant	Influent (raw sewage) Effluent (treated wastewater)	Victoria's Recycled Water [50] Victoria's Recycled Water [50]	90 40	30 000 230	LC–MS/MS [50] LC–MS/MS [50]
Venlafaxine	Antidepressant	Influent (raw sewage) Effluent (treated wastewater)	Victoria's Recycled Water [50] Victoria's Recycled Water [50]	1300 500	4700 1700	LC–MS/MS [50] LC–MS/MS [50]
Diclofenac	NSAID	Influent (raw sewage) Effluent (treated wastewater)	Victoria's Recycled Water [50] Victoria's Recycled Water [50]	170 140	30 000 700	LC–MS/MS [50] LC–MS/MS [50]
Propranolol	Beta‐blocker	Influent (raw sewage) Effluent (treated wastewater)	Victoria's Recycled Water [50] Victoria's Recycled Water [50]	30 30	30 000 110	LC–MS/MS [50] LC–MS/MS [50]

Abrreviations: LC–MS/MS, liquid chromatography–mass spectroscopy/mass spectroscopy; NSAID, nonsteroidal antiinflammatory drugs; WWTP, wastewater treatment plant.

## 3 Biochar for PC Removal From Wastewater

**FIGURE 2:**
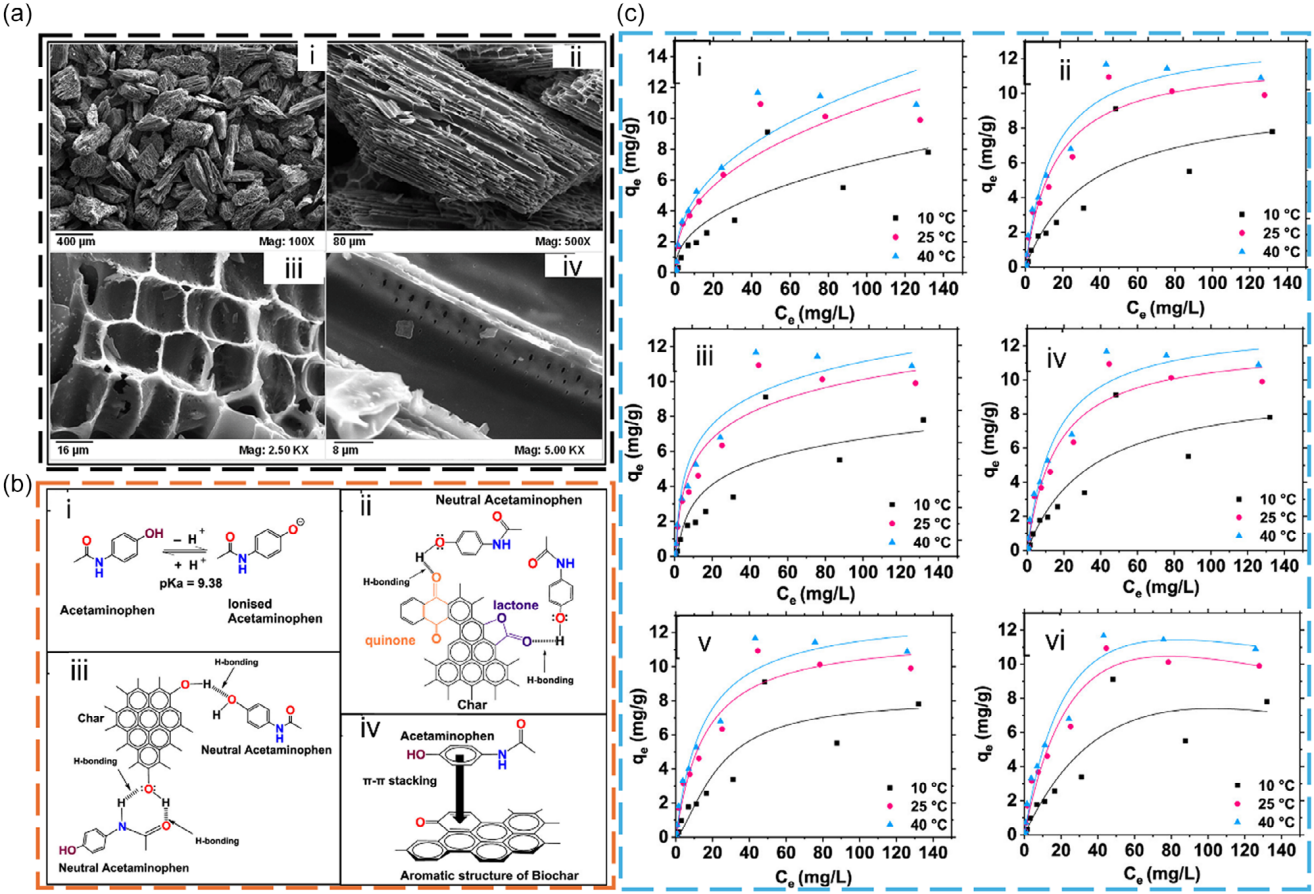
(a) SEM micrographs of *Lantana camara* biochar obtained at 700°C (LB700) at magnifications of (i) 100×, (ii) 500×, (iii) 2.5 KX, and (iv) 5.0 KX; (b) proposed interactions involved in acetaminophen (ACM) sorption: (i) ACM speciation, (ii) hydrogen bonding with quinone and lactone groups on the biochar, (iii) surface hydrogen bonding with phenolic groups, and (iv) π−π interactions between the biochar and ACM (hydrogen bonds are indicated by arrows → in ii and iii); (c) ACM sorption data fitted to (i) Freundlich, (ii) Langmuir, (iii) Temkin, (iv) Redlich−Peterson, (v) Sips, and (vi) Toth isotherm models [pH = 5, LB700 dose = 1 g/L; particle size = 50 − 100 BSS mesh; equilibrium time = 24 h; shaking speed = 100 rpm] at ACM concentrations of 0.5 − 150 mg/L. Experimental data are shown as points, and solid lines represent the corresponding isotherm model fits. The figure is reproduced with permission from [58], copyright @ 2024 American Chemical Society.

The demand for sustainable, eco‐friendly adsorbents has led to biochar‐based materials emerging as a green, effective solution for removing PCs using adsorption methods [29, 30]. An ideal adsorbent should exhibit high adsorption capacity, be cost‐effective to produce, easy to fabricate, and applicable at low adsorbate concentrations. Biochar has gained significant attention for its similarity to AC, featuring a carbon‐rich composition, high stability, porous structure, electrical conductivity, and large surface area. Biochar can be easily produced via thermochemical processes like pyrolysis or gasification from various types of biomass feedstocks [55]. Because of these advantages, biochar has been widely applied to eliminate different types of PCs from an aqueous matrix. The laboratory setup for analyzing the adsorption process of PCs by biochar typically involves batch and column experiments [56]. Key operating parameters include pH, which affects adsorption mechanisms; contact time, which determines kinetic behavior; initial pharmaceutical concentration, influencing adsorption capacity; biochar dose, affecting removal efficiency; and temperature, which impacts the thermodynamics of adsorption [51]. In general, the adsorption of PCs onto biochar involves complex physicochemical processes governed by adsorption kinetics (e.g., pseudo‐first‐order and pseudo‐second‐order models), equilibrium behavior (e.g., Langmuir and Freundlich isotherms), and thermodynamic parameters. Adsorption typically occurs rapidly during the initial stage due to the abundance of available active sites, followed by a slower phase as equilibrium is approached [28]. The overall adsorption mechanism proceeds through several sequential mass transfer steps: (i) transport of adsorbate molecules from the bulk solution to the boundary layer surrounding the adsorbent (bulk diffusion), (ii) movement across the external liquid film to the surface of the biochar (film diffusion), (iii) migration within the pores of the adsorbent (intraparticle or pore diffusion), and (iv) adsorption onto internal active sites [28, 57]. Among these, film diffusion and intraparticle diffusion are often rate‐limiting steps depending on system conditions. The process is commonly well described by the pseudo‐second‐order kinetic model, suggesting that surface interactions play a dominant role in the overall rate. Equilibrium data frequently fit the Freundlich isotherm, indicating heterogeneous multilayer adsorption, although Langmuir behavior may also be observed depending on surface uniformity. Thermodynamic analyses further indicate that adsorption is generally spontaneous (negative Δ*G*) and may be either endothermic or exothermic, reflecting the coexistence of physisorption and chemisorption mechanisms [28]. For instance, a study highlighted the practical use of *Lantana camara*‐derived biochar for ACM from aqueous solutions in a batch mode experimental set‐up [58]. The results of the study are depicted in Figure 2. The results revealed that ACM adsorption was highest and reached a maximum of 4.5 mg/g, which was observed at pH 2 using an LB700 dosage of 1.0 g/L. Sorption efficiency significantly declined for pH values greater than 8, likely because of electrostatic repulsion between deprotonated ACM molecules and the negatively charged surface of biochar [58]. Kinetic studies suggested a pseudo‐second‐order model, whereas thermodynamic results suggested that ACM adsorption was both spontaneous and endothermic. The dominant mechanisms driving ACM sorption onto the biochar included hydrogen bonding, van der Waals forces, pore diffusion, and π–π stacking [58].

The adsorption of PCs onto biochar is driven by various mechanisms, including π–π electron–donor–acceptor (π–π EDA), hydrogen bonding, ionic electrostatic, hydrophobic interactions, and pore filling. Similar adsorption mechanisms, including electrostatic attraction, hydrogen bonding, and π–π interactions, have also been reported in advanced hydrogel‐based adsorbents with tailored internal channel structures, which enhance mass transfer and adsorption selectivity, highlighting the importance of structural design in improving adsorption performance [59]. These processes are fundamentally influenced by the surface functional groups and structure of the biochar such as surface area, pore volume, and porosity, as adsorption is inherently a surface‐dependent phenomenon [60, 61, 62]. In addition, oxygen‐ and nitrogen‐containing functional groups significantly influence biochar's properties and, subsequently, adsorption of PCs. Therefore, the optimization of adsorption operating parameters and employment of biochar with favorable physicochemical properties can help to maximize PCs removal. Desired biochar properties can be tailored to achieve the highest PCs removal through various modification approaches, which are mainly focused to increase surface area, pore volume, optimized surface charge, and improved hydrophilic or hydrophobic characteristics. These improvements significantly enhance their adsorption capacity for PCs, making them more effective for wastewater treatment [62]. Different types of biochar modifications have been discussed in detail in the following sections.

## 4 Modified Biochar: Enhancements for Pharmaceutical Removal

Modifying biochar is essential to enhance their performance and increase their usability in treating polluted water. Various modification techniques, including physicochemical approaches, can be employed to address these challenges [62]. The choice of modification strategy strongly influences the adsorption capacity and mechanisms of biochar toward PCs. Recent studies on advanced adsorbents have further shown that tailoring surface chemistry and structural properties significantly improves both adsorption efficiency and selectivity, highlighting the importance of rational material design in environmental remediation [63].

### 4.1 Physical Modification

Biochar after physical treatment is a scalable and practical method to boost its structural properties, such as pore size, surface area, and texture. Physical modification may involve typical methods like ball‐milling, steam activation, and CO_2_ activation [64, 65]. These methods are simpler, economical, and environmentally friendly than chemical approaches, making them ideal for large‐scale use [66].

#### 4.1.1 Ball Milling

Ball milling is a budget‐friendly approach to produce biochar by reducing grain size (<1000 nm), increasing surface area, and creating surface defects and an enhancement of surface reactive sites relative to raw biochar [67, 68]. The physicochemical properties and functional uses of ball‐milled biochar are significantly influenced by the choice of biochar feedstock and ball milling parameters, such as the milling method, duration, and substrate‐to‐ball weight ratio [30]. Ball‐milling of biochar can be performed in either dry mode or wet‐mode. In wet‐mode, biochar is ground in the presence of a liquid, often water or sometimes organic solvents, whereas in dry‐mode, the grinding of biochar is carried out without any liquid. Both approaches could be useful to alter biochar properties, wet milling can result in finer particles with better heat control where dry mode is more energy efficient and easier to handle. An interesting study compared the physiochemical features of dry‐ and wet‐milled biochar [69]. This work involved the generation of biochar from sawdust by pyrolysis at 600°C for 2 h, labeled as PB. For dry milling, biochar was ground for 2 h (D2) and 12 h (D12), and for wet milling, biochar was mixed with water at a 1:3 ratio and milled for 2 h (W2) and 12 h (W12). The surface area of PB was 154 m^2^/g, while the ball‐milled biochar had higher surface areas: 325 m^2^/g for W2, 334 m^2^/g for W12, 328 m^2^/g for D2, and 360 m^2^/g for D12. These results showed that both types of ball milling enhanced the surface of biochar by 200% compared to raw biochar. In contrast, the average pore sizes decreased from 23.2 nm for PB to 6.37 nm for D2, 6.35 nm for D12, 6.25 nm for W2, and 8.83 nm for W12, indicating the reduction in pore size caused by the breakdown of larger biochar particles. FTIR spectra showed significant changes in functional groups after 12 h of wet ball milling, highlighting the impact of longer milling times [69]. Since it is evident that ball milling can significantly modify biochar properties, the resultant biochar could be utilized for PCs removal. A study demonstrated the application of dry ball‐milled biochar for sulfamethoxazole (SMX) and sulfapyridine (SPY) [69]. Biochar was derived from bamboo, bagasse, and hickory chips at pyrolysis temperatures of 300°C, 450°C, and 600°C. The results showed that ball‐milling introduced a range of functional groups such as –CH_2_, aromatic C = O/C=C, and –CO in the biochar that helped to enhance the removal performance for SMX and SPY [69]. Out of all biochar produced, the 450°C ball‐milled hickory chips biochar showed the highest removal efficiency of 83% for SMX and 90% for SPY [69]. Multiple mechanisms contributed to the adsorption of SMX and SPY on the biochar, encompassing hydrogen bonding, hydrophobic, π–π, and electrostatic interaction [69]. For synthetic water, the biochar showed peak adsorption capacities of 100.3 and 57.9 mg/g for SMX and SPY, respectively. Whereas for real wastewater, adsorption capacities were 25.7 and 58.6 mg/g, SMX and SPY, respectively [69].

Overall, ball‐milling exhibits substantial potential to improve biochar textural properties and subsequently to remove PCs from aqueous solutions. However, certain challenges like high energy consumption should also be considered. Additionally, excessive milling can cause the degradation of the biochar's porous structure, which may decrease its adsorption efficiency. Consequently, applying ball‐milling under optimized conditions, tailored to the specific feedstock characteristics, is essential to maximize benefits while minimizing drawbacks.

#### 4.1.2 Steam and CO_2_ Activation

Steam activation and CO_2_ activation are two other physical modifications that develop highly porous biochar which can be employed for the removal of hydrophilic and hydrophobic PCs. In steam activation, the biochar is exposed to high‐temperature steam (typically 700°C–900°C) under oxygen‐restricted conditions. The steam (H_2_O) reacts with carbon in the biochar to generate CO and H_2_ via water‐gas‐shift reaction. This leads to the oxidation of the carbon and burning away of less organized carbon atoms in the biochar, leading to the generation of mesopores and micropores, which consequently helps to promote the surface area and pore volume. It also introduces oxygen‐containing functional groups (e.g., –OH, –COOH), which may enhance adsorption of PCs [70, 71]. Consequently, steam activated biochar has been used for PCs removal from wastewater. For instance, Rabbat et al. [72] steam‐activated recycled textile biochar and applied for removing ibuprofen from aqueous solutions. The results revealed that steam‐activated biochar showed the surface area of 710 m^2^/g that was almost doubled compared to the pristine biochar of 375 m^2^/g, whereas 160% increase was observed in pore volume, increased from 0.2093 to 0.3363 cm^3^/g [72]. These improved textural properties were possibly due to the generation of pores throughout activation, as carbon atoms are eliminated from the internal carbon network. Subsequently, the modified biochar revealed excellent removal of ibuprofen compared to the raw biochar, achieving the highest adsorption capacity of 54 mg/g [72]. The adsorption of ibuprofen was influenced via different mechanism like π–π interaction, electrostatic interaction, and hydrogen bonding involving different functional groups [72]. Recently, Rong et al. [73] steam‐activated bamboo biochar at different temperatures from 750°C–900°C and investigated their application for SMX elimination from aqueous media. The findings showed that from all biochars, 850°C resulted in biochar with maximum surface area of 1583 m^2^/g compared to 1028 m^2^/g of 750°C. However, a slight decrease was observed in functional groups with an increase in activation temperature. Even though 850°C steam‐activated biochar showed less functional groups, it reached the peak adsorption capacity for SMX, which was 290 mg/g [73].

Similar to steam activation, in CO_2_ activation of biochar, CO_2_ gas is used in place of steam at 700°C–900°C. CO_2_ gas reacts with carbon of biochar via *Boudouard reaction* to produce CO, however, the reaction is slow compared to steam activation, which allows more controlled pore formation in biochar [74, 75]. This approach mainly develops micropores and less mesopores depending on the activation period. In addition, it does not introduce oxygen‐containing functional groups to the same extent as steam activation; hence, the biochar surface remains more hydrophobic [75]. CO_2_ activated biochar is widely applied for the elimination of PCs from water. For instance, a study prepared biochars from different feedstocks like wheat straw pellets, softwood pellets, and peach stone at pyrolysis temperature of 550°C and 700°C, activated them with CO_2_ at 800°C with a flow rate of 1.2 L/min, and tested for the removal of various contaminants, including PCs like chloramphenicol and carbamazepine [74]. The activation significantly increased the surface area for all biochars, especially for wheat straw, it was increased from 16 to 493 m^2^/g for 550°C biochar, and 60–451 m^2^/g for 700°C biochar. Moreover, the overall pore volume was increased from 0.02 to 0.28 cm^3^/g [74]. Consequently, the activated biochars especially from wheat straw and softwood pellets showed improved adsorption capacities for PCs. For example, highest adsorptions of 20.5 and 11.3 mg/g were achieved for carbamazepine and chloramphenicol by activated softwood pellets and wheat straw biochars [74].

Though CO_2_ activation is a feasible approach, studies have shown that steam activation is a more advantageous approach to achieve better biochar properties. For example, a study investigated the physical biochar derived from lignin after activation using H_2_O, CO_2_, and their mixture to produce modified biochar [75]. The results showed that H_2_O was more capable than CO_2_ for pore generation, yielding a specific surface area of 804.1 m^2^/g compared to 516.1 m^2^/g with CO_2_ [75]. Notably, H_2_O promoted mesoporous formation (40% vs. 14% with CO_2_) through enhanced cracking and gasification reactions. However, the combination of CO_2_ and H_2_O showed limited synergistic effects, resulting in a lower surface area (674.8 m^2^/g) due to competitive adsorption, where CO_2_ hindered deoxygenation by H_2_O. In situ IR analysis revealed that CO_2_ suppressed decarbonylation, retaining more C=O groups, while H_2_O promoted their removal. Morphological differences were also observed, with H_2_O activation forming round‐shaped dents and CO_2_ creating round‐edged features on the AC surface [75].

To summarize, steam and CO_2_ activation are effective modification techniques to promote the porosity and adsorption efficiency of biochar for PC removal. Steam activation typically yields higher surface area, mesoporosity, and oxygenated functional groups, promoting its effectiveness for adsorbing diverse PCs, whereas CO_2_ activation promotes controlled micropore development but introduces fewer surface functionalities. Despite their effectiveness, there are some challenges such as high energy requirements, trade‐offs between surface area and functional groups, feedstock variability. Therefore, more research work is required to address these challenges, which could be accomplished through optimization of activation parameters, use of renewable energy, blending of feedstocks, and development of cost‐effective large‐scale activation systems.

### 4.2 Chemical Modification

Chemical modification of biochar is a widely used strategy to promote its physicochemical properties, particularly its surface functionality, reactivity, and selectivity toward specific PCs. This process involves treating biochar with chemical agents that introduce or modify functional groups, alter surface charge, or load reactive elements onto the biochar matrix. Chemical modifications can be further categorized into various types like acid treatment, alkali treatment, oxidant treatment, surface functionalization, metal impregnation, and nanostructured biochar.

#### 4.2.1 Acid Treatment

Biochar modified via acidic treatment like with HCl or H_2_SO_4_ generally introduces O‐containing functional groups like carboxyl, hydroxyl on the biochar surface. These newly formed functional groups might promote electrostatic interactions and hydrogen bonding with polar PCs. In addition, acidic treatment also helps to remove impurities and ash from biochar, which ultimately promotes higher surface area and porosity in biochar. Previous research has reported the successful utilization of acidic modification of biochar for PC removal. For example, a study fabricated pumpkin peels derived biochar with phosphoric acid for ciprofloxacin (CPX) removal from an aqueous medium under varying pH and contact time [76]. The acidic treatment significantly led to a higher surface area of the biochar (690 m^2^/g) and decreased the ash content (6.4%), which could prove high advantageous to enhance CPX adsorption. The results revealed that a highest CPX adsorption capacity of 154 mg/g was achieved at a pH value of 8, reaching equilibrium in 24 h. This is mainly because a change in pH affected biochar surface properties and CPX molecules [76]. At acidic pH (<5.9), biochar surface was positively charged, and CPX molecules were in cationic form, whereas at higher pH, the biochar surface is negatively charged,h and CPX molecules are in anionic form. Therefore, at lower pH, electrostatic repulsions might cause low adsorption capacity. While increasing the pH, there is a high probability that the negatively charged biochar surface can effectively adsorb positively charged CPX molecules, hence contributing to the increased CPX removal. It was also noticed that CPX adsorption enhanced with a rise in temperature, revealing that the adsorption process absorbs heat [76].

Another study modified palm empty bunch‐derived biochar with 2.5 M H_2_SO_4_ and investigated PC adsorption (carbamazepine and ibuprofen) [24]. The results reported that the H_2_SO_4_‐modified biochar achieved an almost 75% higher PC removal rate. Carbamazepine and ibuprofen exhibited maximum adsorption capacities of 51.7 and 38.8 mg/g, respectively. Possible sorption mechanism of PCs on the biochar surface included diffusion through channels, hydrogen bonds, van der Waals, and n–π and π–π stacking interactions [24]. Generally, the presence of other ions (cations/anions) has a pivotal role on the adsorption of PCs. This research evaluated the effect of cations and anions on the adsorption of ibuprofen and suggested that anion species (Cl^−^, NO^3−^, and PO_4_^3−^) might compete for active sites on biochar and thus significantly influenced the adsorption of ibuprofen, as a result, ±20% change in ibuprofen adsorption was noticed. On the other hand, monovalent cations Na^+^ and K^+^ did not affect the adsorption of ibuprofen [24].

To conclude, acidic treatment of biochar using agents like HCl, H_2_SO_4_, or phosphoric acid significantly improves its surface area, porosity, and oxygen‐containing functional groups, thereby enhancing the adsorption of PCs through electrostatic attraction, hydrogen bonding, and π–π interactions. However, adsorption efficiency is strongly influenced by pH, competing ions, and process conditions. Other challenges including pH sensitivity, competition from anions, possible structural damage from strong acids, and environmental concerns may arise from the treatment. These can be addressed through optimized treatment conditions, selective functionalization, milder acid use, and greener acid alternatives.

#### 4.2.2 Alkali Treatment

Similar to acidic activation of biochar, basic solvents have been also proven to enhance biochar properties that further catalyze the elimination of PCs from aqueous sources [77]. Generally, the treatment of biochar with K_2_CO_3_ helps to create more pores and improves the surface area, which enhances the overall adsorption sites on the biochar surface [78]. It has been speculated in earlier studies that K_2_CO_3_ might intercalate through carbon sheets to enlarge the pores, which might cause the creation of mesoporous and microporous structures [78]. Recently, Meseguer et al. [77] studied the performance of K_2_CO_3_‐modified biochar in CPX removal from aqueous solutions. The biochar was derived from barley bagasse generated in the brewing industry and subsequently activated using K_2_CO_3_. The study of adsorption was undertaken in a batch process using 5 g biochar in 100 mL of a 2 mol/L K_2_CO_3_ solution, 25–1000 mg/L concentrations of CPX under varying pH and temperature conditions [77]. The data demonstrated that CPX removal increased with an increase in pH and temperature. However, it was reported that CPX removal at higher pH was attributed to precipitation rather than adsorption, while temperature dependence indicates endothermicity of the adsorption process [77]. The highest adsorption capacity, as determined by different isotherm models, showed variation between 104.9 and 133.4 mg/g. Furthermore, adsorption kinetics analysis suggests that CPX adsorption on biochar involves several steps including CPX diffusion across the boundary layer and within the biochar pores [77]. K_2_CO_3_ activation of biochar for the adsorption of norfloxacin (NOR) was also demonstrated in a separate study, which showed considerably increased adsorption capacity for NOR [79]. It was observed that the biochar produced after the activation with K_2_CO_3_ possessed a very high surface area of 1638 m^2^/g and pore volume of 1.07 cm^3^/g, which achieved a maximum adsorption capacity of 666 mg/g [79]. NOR adsorption on the biochar proceeded spontaneously and released heat, indicating an exothermic process, driven by several forces and pathways like π−π interactions, hydrogen bonding, electrostatic, and pore filling [79].

Egbedina et al. [80] thoroughly compared the performance of acid and base‐activated biochar for PCs removal. For this purpose, firstly, they prepared a hybrid biochar using coconut husk, kaolinite and ZnCl_2_ (labeled as KCB), which was further activated using 50 mL of 2 M HCl (labeled as KCB‐A) and 0.1 M KOH (labeled as KCB‐B). All the prepared biochars were tested in a batch process for the adsorption of CPX and TC by agitating 50 mg of biochar with 10 mL aqueous of either CIP or TC solution (10–150 mg/L) for 2h. Biochar characterization results showed that both acidic and basic treatments significantly increased the carbon content from 43% (in KCB) to 89% in KCB‐A and 82% in KCB‐B. The activation by either of the treatments resulted in structural disorder of the biochar and affected the crystallinity of biochar, since an increase in intensity of x‐ray diffraction peaks was observed. TC and CPX adsorption behavior was quite different with respect to pH, TC adsorption peaked at pH 10, while CPX adsorption was maximum at pH 4. At pH 10, both biochar and TC (as a divalent anionic species) are anticipated to carry a negative charge, so instead of electrostatic repulsion, adsorption was favored, indicating a higher affinity between biochar and TC. At pH < 6, CPX exists as a cation, and it was expected that CPX adsorption would decrease due to electrostatic repulsions between biochar and CPX. However, the adsorption was higher at a low pH of 4, which could be attributed as a result of reduced electrostatic interactions by the hydrophobicity of CPX [80]. Kinetics results further revealed that the adsorption process was governed by multiple forces since monolayer and multilayer adsorption were proceeding simultaneously throughout the system. It was suggested that adsorption of CPX and TC was heterogenous in nature, primarily driven by van der Waal forces, chemisorption, and intraparticle diffusion [80].

To sum up, alkali treatment has several advantages like increasing biochar porosity and basic functional groups on the biochar surface, but the use of alkali solvents could also propose a few challenges, such as their corrosive nature may raise safety concerns. In addition, pH‐dependent adsorption behavior, possible precipitation effects at higher pH, and structural disorder of biochar after harsh chemical activation remain concerns. Therefore, there is a need for finding more environmentally friendly chemicals to reduce environmental impact and tailoring biochar surface chemistry for selective removal of diverse PCs.

#### 4.2.3 Oxidant Treatment

**FIGURE 3:**
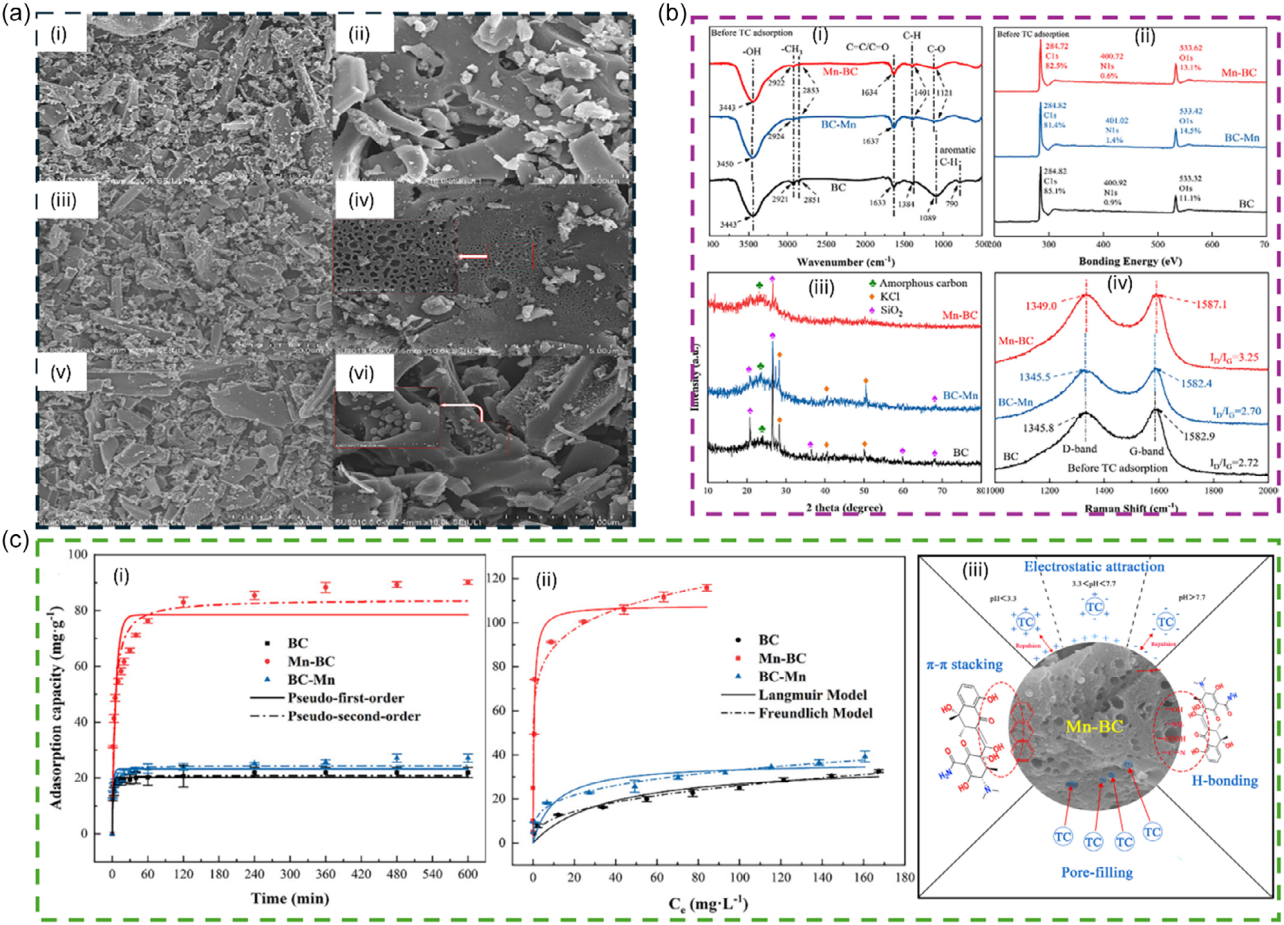
(a): SEM micrographs of control biochar (i, ii), pretreated biochar with KMnO_4_ (iii, iv) and posttreated biochar with KMnO_4_ (v, vi); (b): Biochar characterization, FTIR spectra (i), XPS peak fitting result (ii), XRD images (iii) and Raman spectra (iv) of the biochars; (c) The adsorption kinetic (i) and isotherms (ii) models of TC adsorption onto biochars, and (iii) Schematic diagram of the adsorption mechanism toward TC on Mn‐BC. The figure is reproduced with permission from [81], copyright @ 2023 Elsevier B.V.

Biochar treatment with strong oxidizing agents like KMnO_4_ is another interesting approach that has been employed for PC removal [81, 82, 83]. Since KMnO_4_ is a strong oxidizing agent, it can introduce containing oxygen functional groups (e.g., —COOH, —OH, and —C=O) onto the biochar surface [81]. These groups may improve hydrophilicity and provide additional adsorption sites for a PC via hydrogen bonding and electrostatic interactions. Oxidation can also remove loosely bound organic matter and enhance pore development [81]. This increases the specific surface area and micropore volume, improving physisorption of a PC. Keeping these advantageous effects of KMnO_4_, it was recently used to treat wheat straw biomass (prior to pyrolysis) as well as biochar [81]. The prepared biochars were tested for the extraction of TC from aqueous solution, results are shown in Figure 3 in details. The study suggested that the pretreatment of biomass led biochar performed better for TC adsorption compared to post‐biochar treatment [81]. This was mainly because KMnO_4_ treatment substantially increased the surface area and overall pore volume from control biochar of 70 m^2^/g and 0.0456 cm^3^/g to 728 m^2^/g and 0.489 cm^3^/g, respectively [81]. The treatment also introduced more functional groups and altered crystalline mineral components on the biochar surface. Subsequently, the pretreated biochar achieved a peak adsorption capacity of 108 mg/g, which was nearly 2.7 times higher than posttreated biochar [81]. The adsorption process was determined to be endothermic in nature, and the interactions of TC to the biochar surface were governed by hydrogen binding, π−π interactions, and electrostatic interactions [81]. In a separate study, the performance of KMnO_4_‐treated biochar for TC removal was further enhanced by co‐modification with KOH [83]. In this case, the treated biochar (KOH/ KMnO_4_) showed a surface area of 1525 m^2^/g and total pore volume of 0.85 cm^3^/g. This highly advanced biochar resulted in a maximum TC adsorption capacity of 584 mg/L, attributing to metal complexation, hydrogen binding, pore filling, π−π, and electrostatic interactions [83].

To conclude, oxidant treatment has shown great potential for enhancing PCs' removal by introducing abundant functional groups containing oxygen, improving hydrophilicity, and significantly increasing surface area and porosity, which can help to boost adsorption capacities, driven by several mechanisms, such as electrostatic forces, hydrogen bonding, and π−π interactions. However, challenges include the possible environmental risks of using strong oxidizing agents, cost considerations, and structural instability of biochar under harsh treatments. Therefore, future research should aim at optimizing oxidant dosage, developing safer or greener oxidizing alternatives, and exploring synergistic modifications to balance functional group enrichment with structural durability for scalable and sustainable applications.

#### 4.2.4 Surface Functionalization

Surface functionalization is another commonly used approach for biochar amendment for PCs' removal from aqueous solution [34, 84]. It significantly alters biochar's physicochemical properties, typically improving surface chemistry, porosity, hydrophilicity, and adsorption mechanisms. Recently, Zanli et al. [84] constructed nitrogen‐doped biochar (N—BC) from cocoa shell wastes using urea and further activated it with sodium bicarbonate (NaHCO_3_), assuming to add N‐containing functional groups and improve the microstructure of the biochar. The developed biochar was tested to remove NOR from aqueous solution. Interestingly, the results showed that the combined treatment substantially enhanced the surface area from 26 m^2^/g (control biochar) to 328 m^2^/g and total pore volume from 0.07 to 1.86 cm^3^/g [84]. In addition, the treatment increased the surface nitrogen and oxygen elemental composition by 80% and 20%, respectively, whereas surface functional groups like C—O, C=O, and COO were greatly enhanced in a range of 33‐200%. Subsequently, when the biochar was tested for NOR adsorption, it reached a maximum value of 134 mg/g, where the unmodified biochar could accomplish only 49 mg/g. This significant boost in adsorption can be attributed to outstanding physicochemical properties such as surface area, pore volume, and favorable elemental composition (including N and O‐containing functional groups) of the biochar that provided additional active sites for NOR [84]. Kinetics analysis suggested that the adsorption process primarily followed monolayer chemical adsorption, supported by pore filling, hydrogen bonding, ion exchange, π–π electron–donor–acceptor and electrostatic attractions [84].

In a separate study, Fe/N‐doped biochar was prepared to remove CPX from a mixture of Cu^2+^ [33]. The doped biochar exhibited a surface area of 10 times higher than the undoped biochar, with enhanced pore volume. In this case, the greatest adsorption potential achieved by the doped biochar was 46 mg/g. It was suggested that CPX adsorption on Fe/N‐doped biochar occurred mainly via chemical adsorption (multiple layer chemisorption) [33].

**FIGURE 4:**
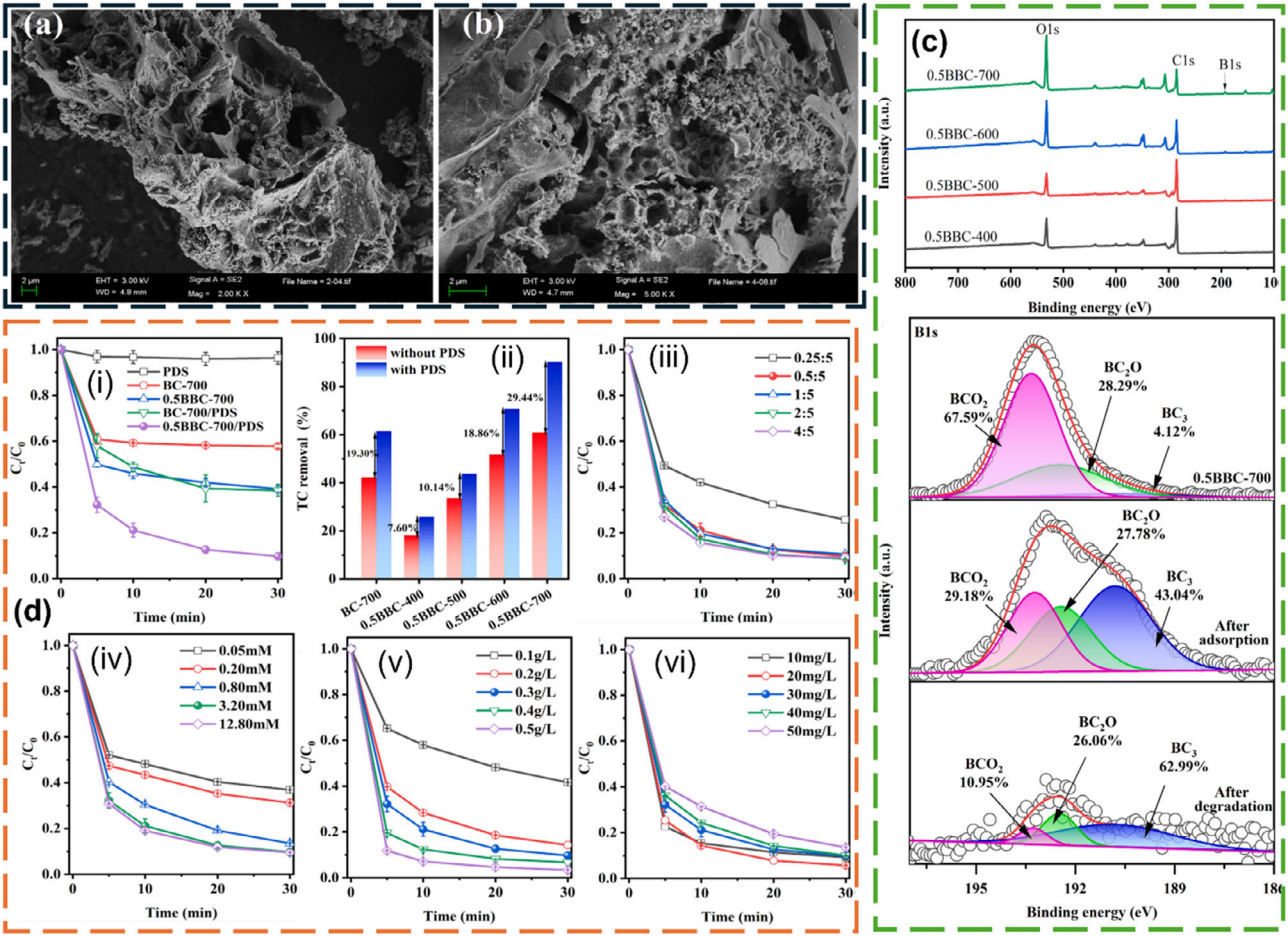
(a, b) SEM images of BC‐700 and 0.5BBC‐700; (c) XPS survey spectra of the B‐doped biochar prepared at different pyrolysis temperatures, along with B1s XPS spectra of 0.5BBC‐700 before reaction, after TC absorption, and after PDS activation; (d) TC removal performance (i) in different systems, (ii) via adsorption and oxidation using 0.5BBC prepared at different pyrolysis temperatures, and the effects of (iii) boric acid dosage, (iv) PDS concentration, (v) activator dosage, and (vi) initial TC concentration on TC removal. Experimental conditions: [TC] = 30 mg/L, [PDS] = 3.2 mM, [activator] = 0.3 g/L, temperature = 25°C, and initial pH unadjusted. The figure is reproduced with permission from [34], copyright @ 2025 Elsevier B.V.

A study by Wang et al. [34] developed a boron‐doped biochar, followed by peroxydisulfate activation for the adsorption of TC. Some important results of the study are shown in Figure 4. Biochar characterization results (Figure 4a‐c) showed successful doping of B on biochar. Further, the authors reported that the incorporation of B introduced additional functional groups like BCO_2_ and BC_2_O on the biochar surface [34]. The treatment also increased the surface area nearly four times and the overall pore volume five times compared to the undoped biochar. Creation of more pores in the biochar can be ascribed to chemical properties of boric acid (salt used for B doping) to serve as an internal template for pore establishment [34]. The biochar contained mesopores (with a pore diameter of 2.5–35 nm) and micropores, exhibiting an average pore diameter of 2.30 nm, allowing for the facile diffusion of TC molecules (1.37 nm). Consequently, the developed biochar showed excellent adsorption capacity for TC compared to undoped biochar, which reached 90% within 30 min. The adsorption process was predicted to take place via commonly known routes like pore filling, ion exchange, H‐bonding, and electrostatic attractions [34].

Surface functionalization has been proven an excellent approach for biochar improvement and PCs' removal, but the doping process could be complex and expensive at the same time. Moreover, potential leaching of dopants and degradation of functional groups are still among major challenges. Hence, future work should prioritize the production of budget‐friendly and environmentally safe functionalization strategies, stabilizing doped biochars to prevent leaching, and tailoring surface chemistry to achieve selective adsorption of target PCs in complex wastewater systems.

#### 4.2.5 Metal Impregnation

Incorporation of metals (e.g., Fe, Mn, Mg, Ca, Zn, Al) onto biochar is an attractive alternative to improve biochar surface reactivity, adsorption capacity, and catalytic properties, which collectively can help to enhance the removal of PCs from wastewater [32]. Metal impregnation introduces metal oxides, hydroxides, or metal‐carbon complexes, which increase active binding sites for PCs [85]. These metals create strong surface complexation and ion exchange interactions with functional groups in PCs, therefore, may enhance adsorption via chelation and coordination bonding (e.g., Fe^3+^‐carboxylate, Mg^2+^‐hydroxyl interactions) [31, 32]. Several studies have demonstrated the employment of metal‐impregnated biochar for enhanced PC adsorption [31, 32, 33]. For example, Zhang et al. [86] prepared Fe/Mn‐loaded biochar for the adsorption of TC. The results showed that Fe/Mn‐biochar achieved approximately seven times enhanced surface area compared to the raw biochar and consequently, showed an increased adsorption capacity of 14 mg/g. The mechanism of the adsorption process of TC on Fe/Mn‐biochar was explained based on pH of the system. Authors reported that at pH < 3, TC mainly existed in the form of TCH_3_^+^, whereas γ‐Fe_2_O_3_ and MnO_2_ (on Fe/Mn‐biochar) hydrolyzed into hydrated minerals of γ‐FeOOH and MnOOH. The OH^−^ ion from γ‐FeOOH and MnOOH is dissociated into H^+^ and transforms the biochar into hydrophilic nature. Therefore, the adsorption of TC onto biochar was predominantly led by H^+^/OH^−^ ion exchange, π–π interactions between the carbon matrix of biochar and TCH_3_^+^ [86]. At pH 3.32, TC mainly existed in the form of TCH_2_^+^, which showed weaker affinity for γ‐FeOOH and MnOOH. However, at pH > 5, TCH_2_^+^ was predicted to be deprotonated into TCH^−^ which could interact with the carbon matrix through electrostatic interactions. At pH > 7.78, TC mainly existed as TC^2−^, showed greater affinity for γ‐FeOOH and MnOOH, thus resulting in enhanced adsorption capacity [86]. Another study by Zhou et al. [87] constructed Fe/Zn‐biochar for the removal of TC, and the data suggested that Fe/Zn‐biochar achieved higher adsorption for TC compared to pristine biochar or either of the individual metal‐loaded biochar. These results were further supported with a fact that Fe/Zn‐biochar occupied the maximum number of TC molecules, which was 0.052 cm^3^ compared to pristine biochar of 0.015 cm^3^ [87]. The adsorption behavior of TC on the biochar was also found changing according to pH and was dominant by H^+^/OH^−^ ion exchange, π–π, electrostatic interactions between carbon matrix and different ions of TC (TCH_3_^+^, TCH_2_^+^, TCH^−^, and TC^2−^) [87].

Al‐loaded biochar was tested in a study by Huang et al. [85] to remove SMX and sulfapyridine (SPY) antibiotics from real wastewater (secondary effluent) in a fixed‐bed column as well as batch sorption mode. Where batch experiments were conducted using 100 mg of biochar, different dosages of Al‐biochar (0, 250, 500 and 1000 mg) were used with 10 mg/L (of SMX and SPY) spiked wastewater. Characterization results confirmed the presence of aluminum oxyhydroxide/oxide and generation of additional oxygen‐containing functional groups in the modified biochar [85]. In batch mode, Al‐biochar achieved maximum adsorption capacities of 1200 and 2200 mg/kg for SMX and SPY, respectively, whereas pristine biochar showed negligible performance for both antibiotics. The excellent sorption capacity of Al‐biochar is attributable to the addition of oxygen‐containing groups and the development of extra active sorption sites. The adsorption mechanism of SMX and SPY on Al‐biochar was analyzed to be primarily driven by hydrophobic, π–π, and electrostatic interactions [85]. On the other hand, in fixed‐bed column, Al‐biochar showed better adsorption capacity for SPY (of 1400 mg/kg) compared to SMX (of 650 mg/kg) [85].

Recently, Yao et al. [31] fabricated MgFe_2_O_4_‐biochar (at 700°C) to study the adsorption of levofloxacin (LFX), using an LFX dose of 100 mg/L and 30 mg of biochar in a batch sorption mode. The results indicated that the addition of MgFe_2_O_4_ decreased the surface area of the biochar, potentially the molecular size of MgFe_2_O_4_ was impermeable into the pores of the biochar or the agglomeration of MgFe_2_O_4_ caused blockage of the pores. However, the total pore volume in the modified biochar significantly increased from 0.04 (pristine biochar) to 0.18 cm^3^/g. Subsequently, the MgFe_2_O_4_‐biochar showed a highest adsorption capacity of 45 mg/g. The adsorption process of LFX onto the biochar was primarily driven by chemisorption, π–π, and electrostatic interactions between functional groups like C—C, O—C=O, and M—O present on the biochar surface and LFX molecules, partially supported by hydrophobic interactions. The adsorption was further enhanced by π–π electron donor–acceptor (EDA) interactions and hydrogen bonding. The O—H groups and graphite‐like structure on MgFe_2_O_4_‐biochar acted as π‐electron donors, while the aromatic ring of LFX served as the π‐electron acceptor [31]. The adsorption was a multilayer and heterogenous process, while thermodynamically it was spontaneous and endothermic in nature [31].

Metal impregnation is an attractive approach to upgrade biochar properties and enhance PCs removal from wastewater, however, preparing metal‐loaded biochar could be a sophisticated process and the leaching of toxic metals could be an ecological concern. Therefore, optimization of metal loading and use of environmentally friendly metals should be prioritized.

#### 4.2.6 Nanostructured Biochar/Nanomaterial Coated Biochar

Nanostructured biochar or a coating of nanomaterials (stated as nanobiochar further in the manuscript) on the biochar can substantially enhance the adsorption of PCs since it significantly increases the surface area as well as the number of micropores and mesopores, thus, the improved pore connectivity allows better diffusion of PCs. Further, the functionalized biochar with nanomaterials could offer a variety of functional groups that can promote affinity for cationic and anionic PCs. This collectively increases the number of sorption sites for enhanced removal of PCs. In the recent decade, multiple studies have undertaken the application of nanobiochar for the adsorption of PCs. For instance, Zhou et al. [88]constructed Fe_3_O_4_/ graphene oxide/citrus peel‐derived biochar using a facile one‐pot hydrothermal process for the adsorption of CPX and sparfloxacin (SPA). The data demonstrated that the nanobiochar achieved a surface area and overall porosity of 1556 m^2^/g and 0.56 cm^3^/g, respectively, which was higher than pristine biochar that had a surface area of 1030 m^2^/g and pore volume of 0.19 cm^3^/g [88]. In addition, the intensity of functional groups like O—H, C=C, O—C=O significantly increased after the addition of graphene oxide [88]. Consequently, the nanobiochar achieved a higher adsorption capacity for both antibiotics, 162 mg/g for CPX and 253 mg/g for SPA. Kinetic behavior and isothermal analysis confirming the adsorption behavior was multilayered and occurred on a heterogeneous surface, predominantly driven by π–π EDA interactions where the graphene surface acted as an electron‐donor, and CPX or SPFX acted as an electron‐acceptor [88]. SPA contains two F atoms in its structure; thus, it is more likely to act as the electron‐acceptor and shows greater affinity for graphene through π–π EDA interactions [88]. The functional groups present on the biochar (O—H, C=C, O—C=O) could interact with either CPX or SPA through H‐bonding. Other forces like hydrophobic interaction and electrostatic interactions were also found participating in the adsorption of CPX and SPA [88].

In a different study, a nanostructured biochar was prepared from pyrolysis of pomegranate peels to investigate the removal of CPX from water [89]. The nanobiochar showed a significant surface area of 1150 m^2^/g, which was 138 times higher than pristine biochar (8.3 m^2^/g). In addition, the nanobiochar contained numerous carboxylic and hydroxyl functionalities on the surface, which could serve as potential sorption sites for CPX [89]. Subsequently, the nanobiochar achieved an excellent removal of CPX, reaching a peak adsorption capacity of 142.8 mg/g whereas the pristine biochar could only achieve an adsorption capacity of 5.9 mg/g. This excellent adsorption by the nanobiochar resulted from the higher surface area along with the presence of enhanced sorption sites [89]. The adsorption of CPX on the biochar was favored by common pathways like π–π interactions, H‐bonding, electrostatic and hydrophobic interactions. To develop π–π interactions, the fluorine group and N‐heteroaromatic ring in CPX molecule may withdraw electrons from the aromatic ring, functioning as π‐electron acceptors [89]. In contrast, the OH groups on the biochar surface may serve as a π‐electron donor. Fluorine atoms of CPX and hydroxyl groups on the biochar surface can interact via H‐bonding [89].

Recently, a study by Kumari et al. [89] constructed a nano‐hybrid structure with biochar embedded with lanthanum ferrite (LaFeO_3_) and employed it for the removal of CPX from wastewater. Authors prepared the biochar from *Murraya koeignii* leaves and encapsulated LaFeO_3_ using one‐step co‐precipitation method. Biochar characterization results confirmed the emergence of mesopores and the presence of oxygen‐rich functional groups in the biochar. The nanobiochar achieved remarkable adsorption capacity of 99 mg/g for CPX [89]. The adsorption of CPX on the nanobiochar was estimated to be monolayer, driven by multiple pathways, including chemisorption, intra particle diffusion, electrostatic and π–π interactions [89].

#### 4.2.7 Combined Approaches

Though an individual process has been widely adopted for the adsorption of PCs, as discussed in previous sections, there are few examples where combined approaches such as anaerobic reactors with metal‐impregnated biochar (adsorption‐biodegradation), and ball‐milling with chemical activation have been experimented to enhance the removal of PCs.

**FIGURE 5:**
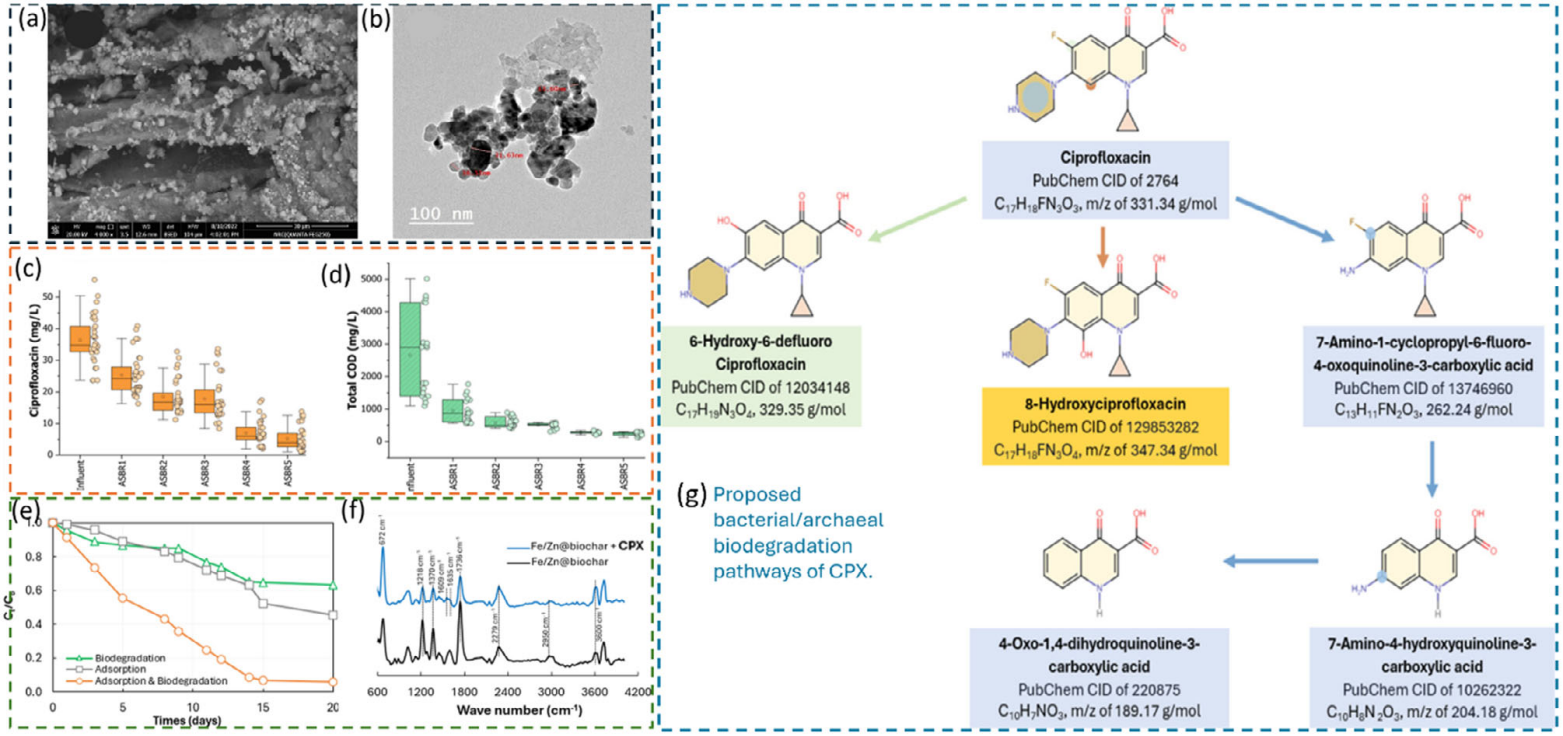
(a) SEM and (b) and TEM of Fe/Zn@biochar; (c) Effect of the Fe/Zn@biochar addition CIP removal efficiency and (d) total COD; (e) Removal mechanism of CPX in batch reactors; (f) FTIR of Fe‐Zn/biochar versus CPX attached on Fe‐Zn/biochar; (g) proposed bacterial/archaeal biodegradation mechanism pathways of CPX. The figure is reproduced with permission from [90], copyright @ 2024 American Chemical Society.

The combined biochar adsorption and biodegradation approach offers a synergistic and efficient method for pharmaceutical removal from water. Biochar rapidly adsorbs PCs, reducing their immediate toxicity and concentration, while also serving as a stable support for microbial communities that can degrade the adsorbed PCs. This dual function enhances removal efficiency, prolongs contact time for microbial action, and potentially regenerates adsorption sites. The combination of adsorption and biodegradation pathways has been proven more effective to remove PCs compared to the individual approach of either adsorption or biodegradation. For example, Tawfik et al. [90] carried out a corroborative study by setting up different batch experiments to understand the effect of individual processes (adsorption and biodegradation) and the synergistic effect of both processes on the removal of CPX in anaerobic sequencing batch reactors (ASBRs). To test the individual adsorption process, 100 mL of wastewater containing 55 mg/L of CPX and 100 mL of water loaded with 223 mg of Fe‐Zn/biochar was used. For the biodegradation process, 100 mL of blank sludge was mixed with 100 mL of wastewater containing 55 mg/g of CPX, whereas for the combined adsorption and biodegradation processes, an additional 100 mg of Fe‐Zn/biochar was added [90]. The results of the study and possible mechanism of CPX degradation by bacteria are depicted in Figure 5. The results revealed that the combined approach outperformed the individual processes from first to the final day of the experiments (see Figure 5e) and achieved a maximum CPX removal efficiency of 94 ± 7%, whereas the individual biodegradation and adsorption process could accomplish maximum CPX removal efficiencies of 35 ± 3% and 55 ± 5%, respectively [90]. Thus, it can be speculated from these results that Fe‐Zn/biochar not only serves as a platform for CPX adsorption but also facilitates direct interspecies electron transfer (DIET) within microbial communities, thereby boosting microbial activity and promoting subsequent biodegradation of CPX. FTIR results shown in Figure 5f (the peak observed around 1736 cm^−1^, associated with the C=O stretching of carbonyl groups on Fe‐Zn/biochar, showing reduction in the peak intensity) suggest that there are strong affinities between CPX molecules and the carbonyl functionalities on the biochar surface. These interactions are likely driven by mechanisms including hydrogen bonding or π–π stacking, where the electron‐dense carbonyl groups engage with the aromatic structures of CPX [90]. The addition of Fe‐Zn/biochar also increased the microbial activity, including improved sludge conductivity and elevated dehydrogenase activity, and contributed to more efficient electron transfer within the syntrophic microbial community [90]. In addition, the study concluded that Fe‐Zn/biochar demonstrated strong stability and can be reused effectively, retaining a peak adsorption efficiency of 99.6% even following four regeneration cycles, which supports its cost‐efficient use in long‐term operations [90]. A different study demonstrated the application of a combined approach of biodegradation and adsorption to increase SMX removal from wastewater using iron‐modified biochar [91]. It was found that iron‐modified biochar successfully enhanced the anaerobic digestion of SMX‐containing wastewater, extracellular respiratory bacteria and hydrogenotrophic methanogen were enriched, whereas antibiotic resistance genes were decreased up to 44%. Consequently, a noticeable increase of 37% was achieved in SMX removal, meanwhile the COD removal was also improved by approximately 23% [91].

Another interesting combined approach used for PCs removal is the application of ball milling and nitrogen doping of biochar. A study demonstrated the application of ball‐milled N‐doped biochar utilized as an adsorbent for the elimination of NOR [92]. The characterization results showed that the combined treatment of ball‐milling and nitrogen doping significantly enhanced the micropore surface area from 14 m^2^/g (pristine biochar) to 107 m^2^/g, whereas the individual treatment of either ball‐milling or N‐doping produced biochars with micropore surface area of 82 and 75 m^2^/g, respectively [92]. Moreover, the micropore volume of 0.061 cm^3^/g was higher than other comparative biochars. Consequently, the treated biochar exhibited a maximum adsorption capacity of 12.53 mg/g, which was four times higher compared to the pristine biochar (2.83 mg/g) [92]. The increased adsorption of NOR was ascribed to the enhancement of H‐bonds, π–π electron donor‐acceptors, and pore filling interactions in the biochar [92].

To conclude, the integration of biochar adsorption with microbial biodegradation presents an effective and synergistic strategy for eliminating PCs from wastewater. The addition of biochar not only improves adsorption through strong molecular interactions but also facilitates microbial electron transfer, leading to accelerated biodegradation. Moving forward, future investigations should concentrate on scaling up this integrated system, examining a wider range of pharmaceuticals, and optimizing biochar modifications to maximize efficiency, reusability, and environmental sustainability.

## 5 Regeneration/Stability of Modified Biochar

Biochar stability in terms of retaining its active sites and composition for effective removal of PCs is highly important for overall economic and environmental feasibility. The long‐term stability and reusability of adsorbent materials are critical factors for their practical application, as they directly influence the economic feasibility and environmental sustainability of wastewater treatment processes [28]. Generally, after the long‐term use of biochar in the process, it becomes saturated with adsorbed PCs, reducing its adsorption capacity, therefore necessitating either disposal or regeneration. Effective regeneration strategies can help minimize the cost and environmental footprint associated with biochar production and disposal, making biochar‐based treatment systems more viable for large‐scale applications. Several methods, including thermal (steam, inert gas, hot water, microwave) and nonthermal (chemical, surfactants, and supercritical) can be used for biochar regeneration. However, it has been documented in the literature that most studies have employed chemical regeneration method for modified biochar targeted for PCs removal. Therefore, only chemical regeneration has been discussed in this section.

Chemical regeneration involving the use of solvents or reagents such as ethanol, acetone, NaOH, or H_2_O_2_ to desorb PCs from the biochar is a widely used approach. This method is generally less energy‐intensive and preserves the physical integrity of the biochar. Out of all the solvents, NaOH has been a favorite choice for the biochar desorption/regeneration process [93]. NaOH generally initiates desorption of PCs through pH‐induced chemical changes that weaken the interactions between biochar and the target compound [93]. NaOH increases the pH of the system, making the biochar surface and PCs (especially acidic ones) turn negatively charged, causing electrostatic repulsion that promotes desorption. In addition, the high pH in the system interrupts hydrogen bonding and weakens π–π interactions between the biochar's aromatic surfaces and PCs. Furthermore, NaOH can clean the biochar surface and reopen blocked pores, facilitating the diffusion of adsorbed PCs [93].

Previous studies suggest that modified biochar is quite stable and demonstrates competent adsorption capacity for PCs. For instance, a study showed that fresh Fe‐Zn/biochar achieved a removal efficiency of 91.6% for TC in the first cycle. Thereafter, the modified biochar was regenerated with 0.1M NaOH, and even after three cycles, the biochar retained 89% removal efficiency for TC [87]. A different study used 0.2 M NaOH for regeneration of Fe–Mn/biochar and tested for CPX removal [94]. The study showed that the biochar retained approximately 80% of removal efficiency after five adsorption–desorption cycles [94].

Ethanol is also a promising solvent that can be used to revive biochar active sites. For instance, CoFe_2_O_4_‐modified biochar was evaluated for its efficiency in removing AMX and achieved 100% removal efficiency after the first cycle [95]. The modified biochar was regenerated with ethanol and tested for five cyclic adsorption–desorption tests. The outcomes suggest that the biochar exhibited excellent stability and achieved a removal efficiency of 92% after the last cycle [95]. This small decline is likely due to the adsorption sites becoming saturated as AMX accumulates on the biochar surface. FTIR results confirmed N‐H, O—H, C=O, and C=C vibrations after the adsorption of AMX on the biochar, indicating their interactions with AMX and biochar surfaces [95]. Ethanol is known to disrupt nonbonding interactions such as Van der Waals forces, π–π bonding, and hydrogen bonds that initially facilitated adsorption [93]. Therefore, it can be speculated that ethanol disrupted these interaction sites between AMX and the biochar surface and promoted desorption of AMX [93, 95]. Another study employed ethanol (combination with ultrasound) for the regeneration of Fe‐Zn/biochar for the removal of TC and CPX [96]. After five adsorption–desorption cycles, individual ethanol treatment could reserve the adsorption capacity of 64% for TC and 67% for CPX in the modified biochar, whereas the combined treatment of ethanol + ultrasound increased the retained adsorption capacities to 85% and 90% for TC and CPX, respectively [96]. This improved performance can be attributed to a synergistic mechanism involving both chemical and physical actions. Ethanol could be responsible for disrupting hydrogen bonding, π–π interactions, and hydrophobic forces between TC and CPX and the biochar surface [96]. Alternatively, ultrasound might have improved ethanol penetration into biochar pores and facilitated the detachment of adsorbed TC and CPX molecules [96, 97, 98, 99, 100, 101, 102].

Table 2 summarizes the performance of modified biochars from all techniques, their operating parameters, the resultant biochar properties after modifications and maximum adsorption capacity and removal efficiencies for selective PCs.

**TABLE 2:** Application of modified biochar for removing PCs. Note: SA means surface area (m^2^/g), PV means pore volume (cm^3^/g), PD means pore diameter (nm).

Raw materials	Pyrolysis condition	Modification method	PC	Unmodified biochar	Modified biochar	Adsorption mechanism	Maximum Adsorption, mg/g	Removal Efficiency	Reference
Bamboo	Subjected to 500°C at 8°C min^−1^ and maintained for 2 h	45 min under 500°C with steam flow (5 mL/min)	Tetracycline	SA, 1.22 PV, 0.01 PD, 1.85	SA, 2.12 PV, 0.02 PD, 4.06	Electrostatic interaction, π–π electron donor–acceptor, and hydrogen bonding	0.144 mmol/g	95.75%	[103]
Recycled textile	Subjected to 550°C at 10°C min^−1^ and maintained for 2 h	60 min under 850°C with steam flow (0.7 mL/min)	Ibuprofen	SA, 375 PV, 0.21	SA, 710 PV, 0.34	Higher developed pores and surface area are behind the greater PCs adhesion	54	>83 %	[72]
Wheat straw	550°C/700°C for 45 min	CO_2_ (1.2 L/min) for 60 min under 800°C	Caffeine, chloramphenicol, carbamazepine, bisphenol A, diclofenac, and triclosan	At 700°C: SA, 59.5 PV, 0.04 PD, 2.87	At 700°C: SA, 452 PV, 0.26 PD, 2.32	Higher developed pores and surface area are behind the greater PCs adhesion	22.8 11.3 15.9 17.5 6.7 20.3	—	[74]
				At 550°C: SA, 15.9 PV, 0.02 PD, 4.87	At 550°C: SA, 493 PV, 0.28 PD, 2.28		22.2 10 16.1 14.2 6.1 18.6	—	
Softwood	550°C/700°C for 45 min	CO_2_ (1.2 L/min) for 60 min under 800°C	Caffeine, chloramphenicol, carbamazepine, bisphenol A, diclofenac, and triclosan	At 700°C: SA, 205 PV, 0.12 PD, 2.29	At 700°C: SA, 485 PV, 0.28 PD, 2.35	Higher developed pores and surface area are behind the greater PCs adhesion	11.3 8.8 20.5 31.6 5.5 30.2	—	Kozyatnyk et al., 2021b)
				At 550°C: SA, 3.4 PV, 0 PD, 3.81	At 550°C: SA, 530 PV, 0.31 PD, 2.34		11.6 6.4 15.4 22.5 4.3 26.5	—	
Peach stones	550°C/700°C for 45 min	CO_2_ for 60 min under 800°C	Caffeine, chloramphenicol, carbamazepine, bisphenol A, diclofenac, and triclosan	At 700°C: SA, 309 PV, 0.16 PD, 2.11	At 700°C: SA, 400 PV, 0.21 PD, 2.11	Higher developed pores and surface area are behind the greater IBP adhesion	1.8 1.6 3.6 6.3 1.4 9.2	–	Kozyatnyk et al., 2021b)
				At 550°C: SA, 316 PV, 0.17 PD, 2.14	At 550°C: SA, 458 PV, 0.25 PD, 2.14		1.6 1.1 1.5 6 1.2 8.4	–	
Date seeds	700°C for 1 h	Superheated steam (1–1.5 kg/h) at a pressure of 1.5 kg/cm^2^ and at 800°C for 1 h	Ibuprofen	—	SA, 513 PV, 0.20	Hydrogen bonding, ion interaction, pH dependence, intraparticle diffusion	10.51	96.24%	[104]
Date seeds	700°C for 1 h	H_3_PO_4_	Ibuprofen	—	SA, 342 PV, 0.13	Hydrogen bonding, ion interaction, pH dependence, intraparticle iffusion	13.87	87.01%	[104]
Wood chips	800°C for 7 h	Wet ball milling	Acetaminophen, ibuprofen, and salicylic acid	SA, 841 PV, 0.38 PD, 22.5	SA, 743 PV, 0.45 PD, 28.5	PCs adsorption onto biochars was strongly influenced by the pore size and electrostatic interactions of the biochars.	196 132 48.8	≈100% ≈90% ≈40%	[105]
*Raw bamboo*	Subjected to 300°C, 450°C, and 600°C for 2 h per temperature.	Ball milling	Sulfamethoxazole, Sulfapyridine	At 300°C: SA, 2	At 300°C: SA, 8.3	Electrostatic, hydrophobic, π–π, and hydrogen bonding interaction	–	≈39% ≈39%	[106]
				At 450°C: SA, 4.7	At 450°C: SA, 299		‐ 57.9	≈70% 89.6%	
				At 600°C: SA, 59	At 600°C: SA, 276		–	≈50% ≈64%	
Hickory chips	Subjected to 300°C, 450°C, and 600°C for 2 h per temperature.	Ball milling	Sulfamethoxazole, Sulfapyridine	At 300°C: SA, 0.8	At 300°C: SA, 5.6	Electrostatic, hydrophobic, π–π, and hydrogen bonding interaction	–	≈40% ≈40%	[106]
				At 450°C: SA, 9.8	At 450°C: SA, 309		100.3 ‐	83.3% ≈83%	
				At 600°C: SA, 221	At 600°C: SA, 270		—	33.4% ≈65%	
Bagasse	Subjected to 300°C, 450°C, and 600°C for 2 h per temperature.	Ball milling	Sulfamethoxazole, Sulfapyridine	At 300°C: SA = 0	At 300°C: SA, 10.8	Electrostatic, hydrophobic, π–π, and hydrogen bonding interaction	—	≈ 45% ≈40%	[106]
				At 450°C: SA, 59	At 450°C: SA, 331		—	≈70% ≈74%	
				At 600°C: SA, 359	At 600°C: SA, 364		—	≈60% ≈70%	
Pumpkin peels	700°C for 1 h	H_3_PO_4_	Ciprofloxacin	—	SA, 690 PV, 0.6 PD, 6.0	—	153.9	77.1%	[107]
Palm empty bunch	Subjected to 450°C at 10°C min^−1^ and maintained for 30 min	H_2_SO_4_	Methyl paraben carbamazepine ibuprofen, and triclosan	SA, 4.05 PV, 0.02	SA, 60.03 PV, 0.54	Hydrogen bonding, channel diffusion, Van der Waals force, and n‐π and π‐π interactions	60.2 51.7 38.8 35.4	80.3% 79.9% 70.2% 74.3%	[24]
Microalgae	Subjected to 600°C at 10°C min^−1^ and maintained for 1 h	H_3_PO_4_ and doped with melamine	Paracetamol	Without doping SA, 324 PV, 0.25	With doping SA, 433 PV, 0.29	Pore‐filling, higher surface area, well‐developed porosity, and a higher number of surface functionalities	120.7	99.9%	[108]
Pomegranate peels	800°C for 2 h	H_3_PO_4_	Ciprofloxacin	—	SA: 1150	Hydrogen bonding, hydrophobic, electrostatic and π–π interaction	142	89.94%	[89]
Coffee grounds	600°C for 2 h	ZnCl_2_	Amoxicillin	SA: 84.64	SA: 520	—	178.57	99.83%	[109]
		H_3_PO_4_			SA: 235		54.645	≈90%	
Walnut shells WS	Subjected to 800°C at 5°C min^−1^ and maintained for 1 h	FeCl_3_ and KOH	Tetracycline	With KOH SA, 1469.42 PV, 2.35	With KOH and FeCl_3_ SA, 1228.01 PV, 2.57	π–π stacking, electrostatic interactions, Lewis acid–base interactions, hydrogen bonding, pore filling, and metal complexation	405.01	89.42%	[110]
Rice husks	Subjected to 800°C at 5°C min^−1^ and maintained for 1 h	FeCl_3_ and KOH	Tetracycline	With KOH SA, 854 PV, 2.31	SA, 766.96 PV, 2.34	π–π stacking, electrostatic interactions, Lewis acid–base interactions, hydrogen bonding, pore filling, and metal complexation	296.06	82.67%	[110]
Cornstalks	Subjected to 800°C at 5°C min^−1^ and maintained for 1 h	FeCl_3_ and KOH	Tetracycline	With KOH SA, 554.32 PV, 2.88	SA, 495.29 PV, 3.34	π–π stacking, electrostatic interactions, Lewis acid–base interactions, hydrogen bonding, pore filling, and metal complexation	175	61.62%	[110]
*Enteromorpha prolifera*	Subjected to 800°C at 5°C min^−1^ and maintained for 2 h	KOH	Sulfamethoxazole	SA: 249	SA: 2172	π–π stacking, partitioning, electrostatic, hydrogen bonding, and Pore‐filling	744	>94%	[111]
Sawdust	Subjected to a 600°C for 2 h, with a heating rate of 10°C min^−1^	FeCl_3_ and ball milling	Tetracycline	SA, 167 PV, 0.07 PD, 1.63	Ball milling SA, 219 PV, 0.30 PD, 5.26	Ion exchange, pore filling, hydrogen bonding, electrostatic interaction, π−π bonds and van der Waals forces	41.08	70%>	[112]
					Fe‐biochar SA, 17.19 PV, 0.13 PD, 28.91		66.91	96.19%	
Pineapple peel	1100°C for 2 h	Iron magnetic nanoparticles	Amoxicillin	SA, 264.9 PV, 0.04 PD:18.8	SA, 164 PV, 0.20 PD, 18.8	—	18.6	≈90%	[113]
*Banana Pseudostem fibers*	Subjected to 350°C and 650°C for 2 h with a 10°C/min heating rate	Impregnated with CoFe_2_O_4_ nanoparticles	Amoxicillin	SA, 19.36 PV, 0.0172	at 350°C SA, 21.87 PV, 0.0204	π–π interactions, and hydrogen bonding, electrostatic	99.99	>90%	[95]
					at 600°C SA, 25.69 PV, 0.0252		99.76	—	

## 6 Life Cycle Assessment & Techno Economic Analysis

LCA is a valuable tool for evaluating the overall environmental performance and sustainability of using biochar/modified biochar to remove PCs from wastewater. It systematically considers the environmental impacts associated with every stage of the biochar life cycle, such as feedstock collection, biochar production through pyrolysis, adsorption process to remove PCs, and finally to the disposal or regeneration of spent biochar. Since the biochar is modified through various processes, evaluation of the modification process should also be considered for their environmental implications. Similarly, techno‐economic analysis (TEA) is a crucial approach for evaluating the financial viability and technical feasibility of using biochar/modified biochar in the removal of PCs from wastewater, which includes energy inputs in all operating parameters in each stage of the process and the cost of materials/reagents used in the holistic process. The combined LCA and TEA analysis could assess the commercial potential of biochar‐based treatment of PCs.

In most of the LCA studies conducted so far, it has been observed that biochar application for PC removal is more environmentally friendly compared to the commercial AC. However, choosing an appropriate biomass feedstock for biochar production is critical. For instance, a LCA study compared 10 environmental impacts of different biochar, such as wood biochar, biosolids biochar, and AC for SMX removal from wastewater [114]. The results demonstrated that wood biochar achieved better environmental benefits compared to AC in 8 out of 10 categories, such as global warming, respiratory effects, noncarcinogenic, which were mainly due to carbon sequestration (0.57 kg CO_2_ eq./kg dry wood) and energy recovery (8.6 MJ heat/kg dry wood) from pyrolytic products [114]. Biosolids biochar showed higher values for all environmental impact categories, which are mainly ascribed to the high energy demand of 14.9 MJ heat/kg to dry biosolids (reducing the moisture content from 77% to 8%) [114]. Compared to unmodified biochar, the modified biochar (e.g., chemical, metal impregnation) requires additional steps, so potential environmental impacts could vary for some categories. Some studies demonstrated environmental impacts of modified biochar. For example, Gallego‐Ramírez et al. [115] carried out LCA evaluation to compare the ecological consequences linked to the creation of biochar from *Pinus patula* and modification of the biochar with Fe metal. The results showed that the environmental impacts were similar for both raw and Fe‐modified biochar except for human toxicity, freshwater ecotoxicity, and ocean ecotoxicity. The higher impacts in toxicity categories can be attributed to airborne and aqueous effluents generated during iron extraction that pose risks to environmental quality, further impacting aquatic and terrestrial organisms [115]. Another LCA study was conducted where acid‐modified biochar was considered to treat 2000 m^3^ of secondary effluent per day, and the results were compared with commercialized granulated activated carbon (GAC) [24]. The results showed that, for several categories, acid‐modified biochar proved a better alternative to GAC. For example, the global warming potential for modified biochar was only 787 kg CO_2_‐eq., which was 200 times lower compared to GAC [24]. Likewise, in terms of human toxicity and reduced fossil depletion, modified biochar proved nearly 13% and 35% lesser compared to GAC. Since acid was used for biochar modification, terrestrial acidification was an interesting parameter; however, for modified biochar, the terrestrial acidification potential was only 2.25 kg SO_2_‐eq., while for GAC, it was at a higher value of 109.4 kg SO_2_‐eq [24].

Biochar is a carbon‐negative material itself since its application can help to reduce CO_2_ levels. However, it should also be economically viable. The economic cost to produce 1 ton of biochar could vary from USD 91 to USD 329 considering labor, raw materials, and transportation costs, whereas commercial AC may cost USD 1100–1700 [116]. However, the cost for modified‐biochar especially for the removal of PC can increase significantly but would be still lesser when compared with AC for the same process. A previous study by Chakraborty et al. [117] suggested that the production cost of wood biochar for the removal of ibuprofen could reach up to USD 3587/ton, which is mainly associated with the posttreatment cost of the biochar that requires to be treated before the final disposal to avoid further contamination [117]. Another study employing acid‐modified biochar for PC removal suggests that that the total production, application, and disposal cost of the modified biochar was USD 1009, whereas for AC, it was USD 37 300. Therefore, application of acid modified biochar could be highly valuable in terms of performance, sustainability and overall cost of the process for PC removal [24].

## 7 Challenges and Way Forward

Biochar modification techniques have shown immense potential in enhancing the adsorption capacity and selectivity of biochar for the removal of PCs from aqueous environments. Each modification method offers unique advantages, but they also present specific challenges that must be overcome to enhance practical applicability and environmental sustainability. Table 3 summarizes major advantages, challenges of all modification techniques, and mechanisms responsible for PC removal. Physical methods such as ball milling, steam activation, and CO_2_ activation are widely used to enhance surface area and porosity. For instance, ball milling reduces particle size and increases surface area, but it may simultaneously damage the porous structure and is highly energy‐intensive, limiting its scalability [67, 68]. Similarly, steam and CO_2_ activation are widely used for developing microporosity and improving surface area [70, 71], however, they require high temperatures and may result in inconsistent pore development, making process control challenging. Chemical modification methods, including acid (HCl, H_2_SO_4_), alkali (NaOH, KOH), and oxidant (H_2_O_2_, KMnO_4_) treatments, further improve adsorption by introducing oxygen‐containing functional groups that enhance hydrogen bonding, electrostatic interactions, and surface reactivity [24, 81]. However, these methods may lead to structural degradation, corrosive handling issues, and potential environmental risks associated with residual chemicals and leaching. In addition to conventional chemical treatments, advanced chemical strategies such as metal impregnation, surface functionalization, and biochar‐based nanocomposites have been widely explored. Metal impregnation (Fe, Mn, Mg) enhances adsorption via complexation, ion exchange, and redox interactions. Despite this, metal‐impregnated biochars may present several environmental and operational limitations, including potential metal leaching during long‐term application, which can lead to secondary contamination and associated ecotoxicological risks. In addition, the stability of immobilized metal species can be significantly affected by varying environmental conditions, particularly changes in pH, which may compromise structural integrity and reduce long‐term performance [118, 119]. In addition, high synthesis costs, operational complexity, and the risk of nanoparticle release limit their practical applicability. Therefore, future research should focus on integrating physical methods with green chemical treatments to reduce energy consumption and improve pore uniformity, optimizing reagent concentrations, developing environmentally benign and scalable modification routes, and enhancing stability through strategies such as encapsulation, chelation, and nanoconfinement to minimize environmental risks. Despite encouraging laboratory‐scale results, the practical application of modified biochar remains limited. Most studies are conducted under controlled laboratory conditions using model PCs, which do not accurately represent real wastewater systems containing diverse and competing contaminants. In addition, laboratory experiments are typically performed at small scales, often limited to a few litters, whereas real‐world applications require treatment of much larger volumes, such as megalitres of wastewater. These limitations create a significant gap between laboratory findings and practical implementation. Therefore, future research should prioritize studies using real wastewater matrices, conduct pilot‐ and industrial‐scale experiments, and evaluate long‐term performance under realistic operating conditions to better assess the feasibility, efficiency, and durability of biochar‐based treatment systems.

**TABLE 3:** Comparison of Biochar Modification Methods for Pharmaceutical Removal.

Method	Advantages	Challenges	Mechanisms of PC removal
Ball milling	‐ Increases surface area ‐ Enhances accessibility of pores and active sites ‐Simple and scalable method with no chemical use	‐ High energy consumption ‐ May collapse porous structure ‐ Limited chemical functionality	‐ Physical adsorption ‐ Increased diffusion rate due to smaller particle size
Steam activation	‐ Increases microporosity and PC surface area‐ Chemical‐free method ‐ Low environmental impact ‐ Compatible with diverse feedstocks	‐ Requires high temperature and pressure ‐ Limited functional group development	‐ Physical adsorption ‐ Pore‐filling and Van der Waals interactions
CO_2_ activation	‐ Produces well‐developed pore structures ‐ Nontoxic and sustainable ‐ Avoids secondary waste generation ‐ Promotes high thermal stability in product	‐ Expensive equipment ‐ Less efficient in forming surface functionalities ‐ May require longer activation times	‐ Physical adsorption ‐ Enhanced diffusion through mesopores/micropores
Acid treatment	‐Adds oxygen‐containing groups (—OH, —COOH, —C=O) ‐ Improves hydrophilicity and PC affinity ‐ Enhances hydrogen bonding capacity ‐ Removes inorganic impurities from biochar	‐ Potential structural degradation ‐ Acid disposal and corrosion concerns ‐ Environmental hazard	‐ Hydrogen bonding ‐ Electrostatic attraction ‐ π–π interactions
Alkali treatment	‐ Enhances surface roughness and porosity ‐ Increases basic functional groups (—OH, —O^‐^) ‐ Improves adsorption of acidic PCs (e.g., ibuprofen) ‐ Helps in ash removal and pore opening	‐ Corrosive nature ‐ Safety concerns ‐ Alters ash content and stability	‐ Ion exchange ‐ Electrostatic interaction with acidic drugs ‐ Base‐catalyzed adsorption
Oxidant treatment	‐ Introduces reactive oxygen species ‐ Boosts surface acidity and hydrophilicity ‐ Enhances redox potential and degradability of certain PCs ‐ Can break down aromatic rings in biochar for more binding sites	‐ Unstable functional groups ‐ Risk of over‐oxidation ‐ Toxic oxidant residues	‐ Redox reactions ‐ Hydrogen bonding ‐ π–π interactions
Surface functionalization	‐ Tailors surface to specific pollutant classes ‐ Enhances selectivity and bonding affinity ‐ Can introduce multiple binding mechanisms (e.g., ion exchange, π–π, covalent bonding)	‐ Complex synthesis ‐ Potential release of reagents ‐ Functional group degradation in environmental conditions	‐ Covalent binding ‐ Ionic bonding ‐ Selective complexation
Metal impregnation	‐ Adds catalytic, magnetic or redox activity ‐ Improves sorption of negatively charged PCs ‐ Supports degradation or transformation of some PCs	‐ Metal leaching ‐ Possible toxicity ‐ Instability under varying pH conditions	‐ Complexation ‐ Precipitation ‐ Redox interactions
Nanomaterial‐biochar composites	‐ Multifunctional adsorbents with high surface area ‐ Enable simultaneous adsorption and degradation ‐ High adsorption efficiency for wide range of PCs	‐ High cost of nanomaterials ‐ Environmental risk due to nanoparticle leaching ‐ Complex and less scalable synthesis processes ‐ May impair biochar biodegradability	‐ Adsorption + catalysis ‐ π–π stacking ‐ Hydrogen bonding and electrostatic forces

The large‐scale deployment of modified biochar technologies requires careful consideration of both economic feasibility and environmental sustainability. High production and modification costs, along with potential environmental impacts associated with chemical usage and energy consumption, remain key challenges. To address these issues, the integration of LCA and TEA is essential for identifying cost‐effective and sustainable production pathways, minimizing environmental footprints, and supporting decision‐making for industrial applications. Furthermore, emerging approaches such as machine learning and computational modeling provide promising tools to optimize biochar design and predict adsorption performance for specific contaminants. By incorporating data‐driven strategies alongside technological innovation, future research can accelerate material optimization and process development. Overall, by combining advances in modification techniques with practical, economic, and environmental considerations, biochar‐based technologies can be more effectively scaled up and applied to address the growing challenge of pharmaceutical pollution in wastewater systems.

## 8 Conclusions

This review concludes that modified biochar is a superior adsorbent for PCs compared to nonmodified biochar, demonstrating excellent removal efficiency from aqueous streams. Physical or chemical modification generally enhances the textural properties of biochar, including pore volume and surface area, and introduces additional functional groups. These changes improve the affinity of biochar for specific PCs by influencing key adsorption mechanisms such as ion exchange, pore filling, hydrogen bonding, hydrophobic interactions, electrostatic interactions, and π–π interactions. Operating parameters, particularly system pH, play a crucial role in determining the surface charge of both the biochar and the PC molecules. Maximum adsorption of a given PC typically occurs at a pH that promotes strong and favorable interactions between them. Regarding regeneration, NaOH has been identified as an effective agent, as it induces pH‐driven chemical changes that weaken the interactions between the biochar surface and the adsorbed PCs. Furthermore, the use of modified biochar is considered more environmentally friendly than commercial AC, with lower environmental impacts such as global warming potential, respiratory effects, and noncarcinogenic risks. It is also economically viable when considering production, application, and disposal costs.

## Author Contributions

**Ebrahim Tangestani**: conception and design of study, acquisition of data, drafting the manuscript; **Ravinder Kumar:** conception and design of study, acquisition of data, analysis and/or interpretation of data, drafting the manuscript, revising the manuscript critically for important intellectual content; **Catherine M. Miller:** revising the manuscript critically for important intellectual conten; **Elsa Antunes:** revising the manuscript critically for important intellectual content.

## Declaration of Generative AI use

The authors declare no generative AI was used for data extraction or prepare any figures/tables.

## Funding

The authors have nothing to report.

## Conflicts of Interest

The authors declare no conflicts of interest.

## Supporting information

Supplementary Material

## Data Availability

The data that support the findings of this study are available from the corresponding author upon reasonable request.
