# Supplementary material for: Recent Advances on Application of Modified Biochar for the Removal of Pharmaceutical Compounds from Wastewater
Source: ChemistryOpen. 2026 May 22;15(6):e70221. doi: 10.1002/open.70221 (PMC13240449; doi:10.1002/open.70221)
Supplement: Supplementary file 1 — Supplementary Material [file OPEN-15-e70221-s001.pdf]

SSupplementary information

Recent Advances on Application of Modified Biochar for the Removal of Pharmaceutical Compounds from Wastewater

Ebrahim Tangestani[1], Ravinder Kumar[1], Catherine M. Miller[2], Elsa Antunes[1*]

[1] College of Science and Engineering, James Cook University, Townsville, Queensland, 4811 Australia

[2] College of Medicine and Dentistry, James Cook University, Cairns, Queensland, 4870 Australia

***Corresponding Author:**

Elsa Antunes

[1]College of Science and Engineering, James Cook University, Townsville, Queensland, 4811 Australia, E-mail: elsa.antunes1@jcu.edu.au

Table S1. Global levels of environmental contamination by commonly used antibiotics.

Pharmaceutical	Source	Location	Average conc.	Max conc	Detection Method
Amoxicillin	Urban sewage	China (Jia et al., 2018)		3380 ng/L	HPLC-MS/MS
	Estuary	China (Lu et al., 2020)		786.40 ng/L	HPLC-MS/MS
	Bay	China (Lu et al., 2020)		90.97 ng/L	HPLC-MS/MS
	Surface water	Merthyr Tydfil, UK (Kasprzyk-Hordern et al., 2007)	39-49 ng/L		UPLC–MS/MS
	Surface water		198-245 ng/l		UPLC–MS/MS
	Surface water	Trefforest Estate, UK (Kasprzyk-Hordern et al., 2007)	56-60 ng/l		UPLC–MS/MS
	Surface water	Cardiff, UK (Kasprzyk-Hordern et al., 2007)		200 ng/L	-
	Effluent	Spain (Elizalde-Velázquez et al., 2016)		900 ng/l	-
	Effluent	Hospital, Spain (Elizalde-Velázquez et al., 2016)		1670 ng/l	-
	Effluent			120 ng/L	HPLC-MS/MS
	Effluent	Urban, Spain (Elizalde-Velázquez et al., 2016)		15 ng/L	HPLC-MS/MS
	Effluent	Palermo , Italy (Castiglioni et al., 2005)		25 ng/L	HPLC-MS/MS
	Effluent	Latina, Italy (Castiglioni et al., 2005)		1600 ng/L	LC-ESI-MS/MS
	Influent	Varese olona, Italy (Castiglioni et al., 2005)		4600 ng/L	LC-ESI-MS/MS
	Effluent	Kenya (Kairigo et al., 2020)		900 ng/l	HPLC-MS/MS
	Influent	Kenya (Kairigo et al., 2020)		6940 ng/l	HPLC-MS/MS
	Raw sewage	Hospital, Australia (Watkinson et al., 2009)		280ng/l	LC-MS/MS
	Effluent	Australia (Watkinson et al., 2009)		30 ng/l	LC-MS/MS
	River			2.7 ng/l	LC-MS/MS

	Influent	Australia (Watkinson et al., 2007)		5.9 ng/l	LC-MS/MS
	Effluent	Brisbane, Australia (Watkinson et al., 2007)		1.3 ng/l	LC-MS/MS
	Hospital	Kumasi, Ghana (Azanu et al., 2018)		6 ng/l	LC-MS/MS
	Irrigation water	Kumasi, Ghana (Azanu et al., 2018)		1.3 ng/l	LC-MS/MS
		Kumasi, Ghana (Azanu et al., 2018)			
		Kumasi, Ghana (Azanu et al., 2018)			
		Kumasi, Ghana (Azanu et al., 2018)			
Ciprofloxacin	Biosolids	Kaikoura (Murdoch)	29 µg/kg		-
	River	Shandong, eastern China (Hanna et al., 2018)	0.867 ng/L	18.8 ng/L	LC-MS/MS
	Wastewater	Shandong, eastern China (Hanna et al., 2018)	8.136 ng/L	99.3 ng/L	LC-MS/MS
	Drinking water		6.205 ng/L	224.4 ng/L	LC-MS/MS
	Effluents	Shandong, eastern China (Hanna et al., 2018)		31 mg/L	HPLC-MS/MS
	Effluent	PCs industries in India (Larsson et al., 2007)		1142.6 ng/L	SPE-LC-MS/MS
	Influent			81 748 ng/L	SPE-LC-MS/MS
	Wastewater	South Africa (Faleye et al., 2019)		15700 ng/L	-
	Effluent	South Africa (Faleye et al., 2019)	96 ng/l	265 ng/l	SPE-LC-MS/MS
	Effluent	Hospital, Switzerland (Carraro et al., 2016)	129 ng/l		UPLC-MS/MS
	River	Spain (Loos et al., 2013)	596.5 ng/l	1168 ng/l	LC-MS/MS
	Influent	Switzerland (De la Cruz et al., 2012)	1209 ng/l	2371 ng/l	LC-MS/MS
	Effluent	Kumasi, Ghana (Azanu et al., 2018)	144.5 ng/l	262ng/l	LC-MS/MS
	Hospital	Kumasi, Ghana (Azanu et al., 2018)	13542.5 ng/l	15733 ng/l	LC-MS/MS

	Irrigation water	Kumasi, Ghana (Azanu et al., 2018)	96.5 ng/l	146 ng/l	LC-MS/MS
	Lake	Kumasi, Ghana (Azanu et al., 2018)	15 ng/l	41 ng/l	LC-MS/MS
	Influent	Kumasi, Ghana (Azanu et al., 2018)		207 ng/l	UPLC-MS/MS
	Effluent	Uganda (Nantaba et al., 2020)		313 ng/l	HPLC–QTRAP- MS
	River	Tunisia (Moslah et al., 2018)		109 ng/l	HPLC–QTRAP- MS
	Influent	Ebro river region, Spain (Gros et al., 2010)	5524 ng/l	13625 ng/l	LC–QTRAP–MS
	Effluent	Ebro river region, Spain (Gros et al., 2010)	2378 ng/l	5692 ng/l	LC–QTRAP–MS
	Lake	Alcala de Henares, Spain (Rosal et al., 2010)	7.3 ng/l	120 ng/l	LC–MS/MS
		Alcala de Henares, Spain (Rosal et al., 2010)			
		Africa (Nantaba et al., 2024)			
Tetracycline	Influent	Municipal WWTPs, Gulf, Iran (Kafaei et al., 2018)		0.0111µg/L	HPLC–ESI-MS/MS
	Influent			0.144µg/L	HPLC–ESI-MS/MS
	Effluent	Hospital WWTPs, Gulf, Iran (Kafaei et al., 2018)		0.15µg/L	UHPLC-ESI-MS-MS
	Influent	Guangdong, China (Zhang et al., 2018)		0.18µg/L	UHPLC-ESI-MS-MS
	River	Guangdong, China (Zhang et al., 2018)		54ng/L	HPLC-MS/MS
	Lake	Drewe River, Poland (Harnisz et al., 2015)		101ng/L	UPLC-ESI-MS-MS
	Sludge	Hanoi, Vietnam (Tran et al., 2019)		62µg/kg dw	UHPLC-ESI-MS-MS
	River	Brazil (Bisognin et al., 2021)		11.16 ng/L	UPLC-ESI-MS/MS
	Influents	Yangtze, China (Wang et al., 2019)	16.15 ng/l	32.3 ng/l	-
	Effluents	Portugal (Patel et al., 2019)	11.4 ng/l	22.8 ng/l	-

	Effluent	Portugal (Patel et al., 2019)	0.22 µg/l	0.37 µg/l	-
	Surface water	USA (Al-Riyami et al., 2018)	0.11 µg/l		LC-MS/MS
	Ground water	USA (Mackie et al., 2006)	>0.5 ug/l		LC-MS/MS
	Surface water	USA (Mackie et al., 2006)	2.7 ug/l	4.2 µg/l	-
	Surface water	Germany (Borghi and Palma, 2014)		0.11 µg/l	-
	Agriculture water	UK (Borghi and Palma, 2014)	180 ng/l		-
	Influents	Thailand (Lundborg and Tamhankar, 2017)		48 µg/l	LC-MS/MS
	Effluents	USA (Batt et al., 2007)		0.41 µg/l	HPLC-UV
	Effluents	USA (Khan et al., 2010)		3170 µg/l	UPLC-MS/MS
	River	PCs WWTPs, China (Hou et al., 2016)	20.5 ng/l	30 ng/l	LC-MS/MS
	Influent	Kumasi, Ghana (Azanu et al., 2018)	106 ng/l	199 ng/l	LC-MS/MS
	Effluent	Kumasi, Ghana (Azanu et al., 2018)	17.5 ng/l	24 ng/l	LC-MS/MS
	Hospital	Kumasi, Ghana (Azanu et al., 2018)	87 ng/l	30 ng/l	LC-MS/MS
	Irrigation water	Kumasi, Ghana (Azanu et al., 2018)	13.5 ng/l	16 ng/l	LC-MS/MS
	Lake	Kumasi, Ghana (Azanu et al., 2018)	9.5 ng/l	70 ng/l	LC-MS/MS
	Effluent	Uganda (Nantaba et al., 2020)		340 ng/l	HPLC–QTRAP- MS
	Lake	Ebro river region, Spain (Gros et al., 2010)	1.6 ng/l	31 ng/l	LC–MS/MS
		Africa (Nantaba et al., 2024)			
Trimethoprim	Marine sediments	Auckland, New Zealand (Murdoch)	0.23 µg/kg		LC-MS/MS (Stewart, 2013)
	Biosolids	Kaikoura, New Zealand (Murdoch)	5 µg/kg		-
	Effluent	Kenya (Kairigo et al., 2020)		300 ng/L	LC-ESI-MS/MS

	Influent	Kenya (Kairigo et al., 2020)		5600 ng/L	LC-ESI-MS/MS
	Effluent	South Wales (UK) (Loos et al., 2013)	178 ng/l	800 ng/l	SPE-LC-MS/MS
	Influent	Spain (Bueno et al., 2012)	0.72 µg/l	0.13 µg/l	LC-MS/MS
	Effluent	Spain (Bueno et al., 2012)	0.66 µg/l	0.13 µg/l	LC-MS/MS
	River	Japan(Murata et al., 2011)	0.04 µg/l		LC-MS/MS
	River	UK (Roberts and Thomas, 2006)	0.02 µg/l		HPLC–ESI-MS/MS
	Effluent	Switzerland (De la Cruz et al., 2012)	131 ng/l		UPLC-MS/MS
	River	Kumasi, Ghana (Azanu et al., 2018)	418.5 ng/l	820 ng/l	LC-MS/MS
	Influent	Kumasi, Ghana (Azanu et al., 2018)	851.5 ng/l	1668 ng/l	LC-MS/MS
	Effluent	Kumasi, Ghana (Azanu et al., 2018)	13 ng/l	255 ng/l	LC-MS/MS
	Hospital	Kumasi, Ghana (Azanu et al., 2018)	2460 ng/l	4826 ng/l	LC-MS/MS
	Irrigation water	Kumasi, Ghana (Azanu et al., 2018)	58.5 ng/l	98 ng/l	LC-MS/MS
	Lake	Uganda (Nantaba et al., 2020)	11 ng/l	89 ng/l	LC-MS/MS
	Downstream	South Africa (Archer et al., 2017)	898.7 ng/l		UPLC-MS
	Upstream	South Africa (Archer et al., 2017)	383 ng/l		UPLC-MS
	Effluent	Ebro river region, Spain (Gros et al., 2010)		463 ng/l	HPLC–QTRAP- MS
	River	Ebro river region, Spain (Gros et al., 2010)		114 ng/l	HPLC–QTRAP- MS
	Influent	Alcala de Henares, Spain (Rosal et al., 2010)	104 ng/l	197 ng/l	LC–QTRAP–MS
	Effluent	Alcala de Henares, Spain (Rosal et al., 2010)	99 ng/l	148 ng/l	LC–QTRAP–MS
	Lake	Africa (Nantaba et al., 2024)	3.9 ng/l	22 ng/l	LC–MS/MS

Sulfamethoxazole	Biosolids	Kaikoura, New Zealand (Murdoch)	15 µg/kg		-
	River	Shandong, eastern China (Hanna et al., 2018)	1.301 ng/L	13 ng/L	LC-MS/MS
	Wastewater	Shandong, eastern China (Hanna et al., 2018)	1.389 ng/L	20.9 ng/L	LC-MS/MS
	Drinking water		2.090 ng/L	18.6 ng/L	LC-MS/MS
	Effluent	Shandong, eastern China (Hanna et al., 2018)		8500 ng/L	LC-ESI-MS/MS
	Influent	Kenya (Kairigo et al., 2020)		49300 ng/L	LC-ESI-MS/MS
	Wastewater	Kenya (Kairigo et al., 2020)		25300 ng/L	-
	Wastewater	Hospital, South Korea (Carraro et al., 2016)		2935 ng/L	LC-MS/MS
	River	Domestic, China (Yuan et al., 2016)		1.45 mg/kg	UPLC- QTOF-MS
	Effluent	South Africa (Ohoro et al., 2021)	142 ng/L	1174 ng/l	SPE-LC-MS/MS
	Effluent	Spain (Loos et al., 2013)	578 ng/l		UPLC-MS/MS
	River	Switzerland (De la Cruz et al., 2012)	1437 ng/l	2861 ng/l	LC-MS/MS
	Influent	Kumasi, Ghana (Azanu et al., 2018)	3718 ng/l	7194 ng/l	LC-MS/MS
	Effluent	Kumasi, Ghana (Azanu et al., 2018)	211.5 ng/l	320 ng/l	LC-MS/MS
	Hospital	Kumasi, Ghana (Azanu et al., 2018)	2952.5 ng/l	3590 ng/l	LC-MS/MS
	Irrigation water	Kumasi, Ghana (Azanu et al., 2018)	33.5 ng/l	56 ng/l	LC-MS/MS
	Surface water	Kumasi, Ghana (Azanu et al., 2018)		11.8 ng/l	SPE-LC-MS/MS
	Surface water	River Ledave, Slovenia (Klančar et al., 2018)		6.25 ng/l	SPE-LC-MS/MS
	Surface water			1.2 ng/l	SPE-LC-MS/MS
	Lake	River Kokra, Slovenia (Klančar et al., 2018)	5.8 ng/l	5600 ng/l	LC-MS/MS
	Influent	River Zbilje, Slovenia (Klančar et al., 2018)		198 ng/l	UPLC-MS/MS

	Effluent	Uganda (Nantaba et al., 2020)		265 ng/l	UPLC-MS/MS
	Downstream	Tunisia (Moslah et al., 2018)		1013.2 ng/l	UPLC-MS
	Upstream	Tunisia (Moslah et al., 2018)		757.4	UPLC-MS
	Effluent	South Africa (Archer et al., 2017)		620 ng/l	HPLC–QTRAP- MS
	River	South Africa (Archer et al., 2017)		36 ng/l	HPLC–QTRAP- MS
	Influent	Ebro river region, Spain (Gros et al., 2010)	279 ng/l	530 ng/l	LC–QTRAP–MS
	Effluent	Ebro river region, Spain (Gros et al., 2010)	231 ng/l	370 ng/l	LC–QTRAP–MS
	Influent	Alcala de Henares, Spain (Rosal et al., 2010)	88.1 ng/l		SPE-UHPLC-MS/MS
	Effluent	Alcala de Henares, Spain (Rosal et al., 2010)	87.2 ng/l		SPE-UHPLC-MS/MS
	Lake		1.3 ng/l	8.2 ng/l	LC–MS/MS
		Saudi Arabia (Mostafa et al., 2023)			
		Saudi Arabia (Mostafa et al., 2023)			
		Africa (Nantaba et al., 2024)			

Table S2. Global levels of environmental contamination by commonly used antidepressants.

Pharmaceutical	Source	Location	Average conc.	Max conc	Detection Method
Sertraline (SER)	Influent	Municipal, Germany (Schlüsener et al., 2015)	49 ng/L		-
	Effluent		9 ng/L		-
	Influent	Municipal, Germany (Schlüsener et al., 2015)	31.6–114 ng/L		HPLC-MS/MS
	Effluent	River, USA (Subedi and Kannan, 2015)	15.7–88.3 ng/L		HPLC-MS/MS
	Influent		0.77–3.11 ng/L		HPLC-MS/MS
	Effluent	River, USA (Subedi and Kannan, 2015)	5.9–7.38 ng/L		HPLC-MS/MS
	Influent	Municipal, Mexico (Estrada-Arriaga et al., 2016)	417 ng/L		UHPLC-MS/MS
	Effluent	Municipal, Mexico (Estrada-Arriaga et al., 2016)	25 ng/L		UHPLC-MS/MS
	Influent		79.5 ng/L		UHPLC-MS/MS
	Effluent	Municipal, Brazil (Pivetta et al., 2020)	94.3 ng/L		UHPLC-MS/MS
	Influent	Municipal, Brazil (Pivetta et al., 2020)		997 ng/l	-
	Effluent	Hospital, Greece (Kosma et al., 2020)		1930 ng/l	-
	Fresh water	Hospital, Greece (Kosma et al., 2020)		75 ng/l	-
	Influent	across geographic regions (Mole and Brooks, 2019)	3.25-6.36 ng/l		LC-MS/MS
	Effluent	across geographic regions (Mole and Brooks, 2019)	4.19-115.3 ng/l		UHPLC-MS/MS
	Surface water			0.24 ng/l	SPE-LC-MS/MS
	Hospital (sewage)	across geographic regions (Mole and Brooks, 2019)		549 ng/l	HPLC
	Influent	Huangpu River, Shanghai (Ma et al., 2018)		119 ng/l	HPLC
	Effluent			26 ng/l	HPLC

	Effluent	Huangpu River, Shanghai (Ma et al., 2018)		1 µg/l	LC-MS/MS
	Surface water	River Ledave, Slovenia (Klančar et al., 2018)	16-20 ng/l		UHPLC-MS/MS
	Effluent	Slovenia (Gornik et al., 2020)	09-2 ng/l		LC-MS/MS
	Surface water	Slovenia (Gornik et al., 2020)	0.4 ng/l	4.64 ng/l	-
	Effluent	Slovenia (Gornik et al., 2020)		80 ng/l	-
	Surface water	Niagara river, USA (Arnnok et al., 2017)		8.9 ng/l	-
	Effluent	Japan (Duarte et al., 2020)	2.2-37.5 ng/l		-
	Surface water	Norway (Metcalfe et al., 2010)		49 ng/l	GC/MS
	Surface water	Turkey (Marrone et al., 1966)		5.4 ng/l	GC/MS
	Surface water	USA (Thörnqvist et al., 2019)	23.3-23.6 ng/l	23.6 ng/l	-
	Surface water	USA (Huggett et al., 2003)	16.01 ng/l	40.3 ng/l	HPLC-UV
	Surface water	USA (Huggett et al., 2003)		17 ng/l	-
	Effluent	USA (Fitzsimmons et al., 2001)	16 ng/l		-
	Effluent	Portugal (Rúa-Gómez and Püttmann, 2012)	8 ng/l		-
	Effluent	Portugal (Nakamura et al., 2008)	8-90 ng/l		-
		Canada (Brunello et al., 2003)			
		Canada (Naderi et al., 2016)			
		Canada (Naderi et al., 2016)			
		Oslo, Norway (Kallenborn et al., 2018)			

		Troms, Norway (Kallenborn et al., 2018)			
Venlafaxine (VFX)	Influent	Lis river, Portugal (Paíga et al., 2016)	11.5 ng/L	39.4 ng/L	UHPLC-MS/MS
	Effluent	Lis river, Portugal (Paíga et al., 2016)	166 ng/L	327 ng/L	UHPLC-MS/MS
	Effluent	Spain (Loos et al., 2013)	119 ng/l		SPE-LC-MS/MS
	Influent	Huangpu River, Shanghai (Ma et al., 2018)	31.09-82.02 ng/l	82.02 ng/l	UHPLC-MS/MS
	Effluent	Huangpu River, Shanghai (Ma et al., 2018)	49.94-132.04	132.04 ng/l	UHPLC-MS/MS
	Effluent			400 µg/l	LC-MS/MS
	Surface water	River, Ecuador (Voloshenko-Rossin et al., 2015)		3.01 ng/l	SPE-LC-MS/MS
	Surface water	River Ledave, Slovenia (Klančar et al., 2018)		1.02 ng/l	SPE-LC-MS/MS
	Surface water			0.09 ng/l	SPE-LC-MS/MS
	Surface water	River Paka, Slovenia (Klančar et al., 2018)		0.08 g/l	SPE-LC-MS/MS
	Surface water	River Savinja, Slovenia (Klančar et al., 2018)		2653 ng/l	-
	Surface water			901 ng/l	-
	Effluent	River Zbilije, Slovenia (Klančar et al., 2018)	808 ng/l		-
	Surface water	Canada (Fraher et al., 2016)	244.11 ng/l	573 ng/l	HPLC-UV
	Surface water	Canada (Naderi et al., 2016)		122 ng/l	-
	Surface water	Canada (Naderi et al., 2016)		100 ng/l	LC-MS
	Effluent	Canada (Brunello et al., 2003)	220 ng/l		LC-MS
	Surface water	Germany (Bataineh and Daradka, 2007)		197 ng/l	-
	Surface water	Germany (Silva et al., 2012)	310 ng/l		HPLC-MS

	Effluent	Germany (Silva et al., 2012)		410 ng/l	-
	Surface water	Italy (Fick et al., 2009)		1000 ng/l	GC/MS
	Surface water	USA (Melvin, 2017)	73.3-359 ng/l		-
	Effluent	USA (Thörnqvist et al., 2019)	188-690 ng/l		-
	Surface water	USA (Fitzsimmons et al., 2001)		71.9 ng/l	-
	Effluent	USA (Huggett et al., 2003)		269.6 ng/l	-
	Surface water	USA (Huggett et al., 2003)	0.9-85.4 ng/l		-
	Effluent	UK (Holzschuh et al., 2001)	94.9 ng/l	285.1 ng/l	-
	Surface water	UK (Holzschuh et al., 2001)		5.6 ng/l	LS-MS/MS
	Surface water	UK (Schultz, 2007)	1.98 ng/l	7.68 ng/l	-
	Downstream	UK (Schultz, 2007)	94.6 ng/l		UPLC-MS
	Upstream	Sweden (Koba et al., 2018)	35.4 ng/l		UPLC-MS
		Turkey (Marrone et al., 1966)			
		South Africa (Archer et al., 2017)			
		South Africa (Archer et al., 2017)			

Table S3. Global levels of environmental contamination by commonly used beta-blocker.

Pharmaceutical	Source	Location	Average conc.	Max conc	Detection Method
Atenolol (ATO)	Lake	Uganda (Nantaba et al., 2020)	85 ng/l	380 ng/L	LC-MS/MS
	Influent	Tunisia (Moslah et al., 2018)		2198 ng/L	UPLC-MS/MS
	Effluent	Tunisia (Moslah et al., 2018)		1244 ng/l	UPLC-MS/MS
	Downstream	South Africa (Archer et al., 2017)	272 ng/L		UPLC-MS
	Upstream	South Africa (Archer et al., 2017)	156 ng/l		UPLC-MS
	Influent	South Africa (Kanama et al., 2018)	4410 ng/L	8340 ng/L	LC-MS/MS
	Effluent	South Africa (Kanama et al., 2018)	1190 ng/l	3220 ng/l	LC-MS/MS
	River	South Africa (Mashile and Nomngongo, 2021)		4.92 ng/L	-
	Wastewater	South Africa (Mashile and Nomngongo, 2021)		28.9 ng/L	-
	Groundwater			0.729 ng/L	HPLC-MS
	Influent	Republic of Korea (Lee et al., 2019)	0.0047 µg/l	361 ng/L	LC-QqQ-MS
	Surface water	Pakistan (Ashfaq et al., 2019)	3.44-241 ng/l		LC-MS/MS
	Surface water	France (Vulliet and Cren-Olivé, 2011)	4-32 ng/l		HPLC-MS/MS
	Surface water	Italy (Calamari et al., 2003)	80-1600 ng/l		LC-MS/MS
	Effluent	Mexico (Rivera-Jaimes et al., 2018)	170-381 ng/l		LC-MS/MS
	Effluent	India (Anumol and Snyder, 2015)	40-440 ng/l		LC-MS/MS
	Effluent	France (Chiffre et al., 2016)	40-1180 ng/l		LC-MS/MS
	Effluent	Finland (Vieno et al., 2006)	64-474 ng/l		HPLC-MS/MS
	Effluent	Finland (Vieno et al., 2007)	86-611 ng/l		HPLC-MS/MS

	Effluent	Italy (Al Aukidy et al., 2012)			LC-QqQ-MS
	Groundwater	New Zealand (Kumar et al., 2019)	5.5 ng/l	6.2 ng/l	LC-MS/MS
	Groundwater	Germany (Vulliet and Cren-Olivé, 2011)	1140 ng/l		HPLC-MS/MS
	Effluent	France (Reh et al., 2013)		2110 ng/l	UPLC-MS/MS
	Surface water	Spain (Biel-Maeso et al., 2018)	1-280 ng/l	122 ng/l	LC-MS/MS
	Surface water	Turkey (Aydin and Talinli, 2013)			LC-MS
	Surface water	Sweden (Bendz et al., 2005)	280 ng/l	560 ng/l	MS
	Effluent	UK (Kasprzyk-Hordern et al., 2009)		690 ng/l	SPE-LC-MS/MS
	Effluent	Portugal (de Jesus Gaffney et al., 2017)	330-1530 ng/l	208 ng/l	HPLC-MS/MS
	Effluent	USA (Lara-Martín et al., 2014)			HPLC-MS
	Effluent	Switzerland (Alder et al., 2010)	1060 ng/l	160 ng/l	LC-MS
	Effluent	Sweden (Bendz et al., 2005)		1370 ng/l	LC–QqLIT- MS
	Effluent	Spain (Gros et al., 2008)	68-138.5		HPLC-MS/MS
	Effluent	Netherlands (Oosterhuis et al., 2013)	970 ng/l		LC-MS/MS
	Effluent	Australia (Roberts et al., 2016)	2225 ng/l	1866 ng/l	LC-MS/MS
	Surface water	Greece (Papageorgiou et al., 2016)			LC–QqLIT- MS
	Surface water	Spain (Valcárcel et al., 2011)		0.49 ng/l	LC-MS/MS
	Surface water	Portugal (Rivera-Jaimes et al., 2018)		110 ng/l	HPLC-MS
	Effluent	Switzerland (Alder et al., 2010)		1094 ng/l	HPLC–QTRAP- MS
	River	Ebro river region, Spain (Gros et al., 2010)		86 ng/l	HPLC–QTRAP- MS
	Influent	Ebro river region, Spain (Gros et al., 2010)	1197 ng/l	2432 ng/l	LC–QTRAP–MS

	Effluent	Alcala de Henares, Spain (Rosal et al., 2010)	1025 ng/l	2438 ng/l	LC–QTRAP–MS
	Influent		91.1 ng/l		SPE-UHPLC-MS/MS
	Effluent	Alcala de Henares, Spain (Rosal et al., 2010)	94.5 ng/l		SPE-UHPLC-MS/MS
	Lake	Saudi Arabia (Mostafa et al., 2023)	4.1 ng/l	20 ng/l	LC–MS/MS
		Saudi Arabia (Mostafa et al., 2023)			
		Africa (Nantaba et al., 2024)			
Propranolol (PRO)	Biosolids	Kaikoura, New Zealand (Murdoch)	114 µg/kg		-
	Wastewater	Tunisia (Moslah et al., 2018)		2198 ng/L	UPLC-MS/MS
	Wastewater	South Africa (Mashile and Nomngongo, 2021)		20.6 ng/L	-
	River water			2.05 ng/L	-
	Groundwater	South Africa (Mashile and Nomngongo, 2021)		0.0980 ng/L	HPLC-MS
	Influent	Republic of Korea (Lee et al., 2019)		1.15 g/L	LC-QqQ-MS
	Surface water	Pakistan (Ashfaq et al., 2019)	1.2 ng/l		LC-MS/MS
	Surface water	France (Vulliet and Cren-Olivé, 2011)	0.13 ng/l	1.89 ng/l	LC-MS/MS
	Surface water	Portugal (Rivera-Jaimes et al., 2018)	46 ng/l	178 ng/l	LC–QqLIT- MS
	Surface water	Spain (Valcárcel et al., 2011)		40 ng/l	MS
	Surface water	UK (Kasprzyk-Hordern et al., 2009)		561 ng/l	LC-MS/MS
	Surface water	Turkey (Aydin and Talinli, 2013)		50 ng/l	LC-MS
	Effluent	Sweden (Bendz et al., 2005)	32.2-79 ng/l		LC-MS/MS
	Effluent	Australia (Roberts et al., 2016)	416 ng/l	1111ng/l	GC-MS
	Effluent	France (Miège et al., 2006)		17.5 ng/l	LC-MS/MS

	Effluent	France (Chiffre et al., 2016)		91 ng/l	LC-MS/MS
	Effluent	Greece (Papageorgiou et al., 2016)	410-1480 ng/l		HPLC-MS/MS
	Effluent	Netherland (Oosterhuis et al., 2013)	170 ng/l	420 ng/l	SPE-LC-MS/MS
	Groundwater	Portugal (de Jesus Gaffney et al., 2017)	1.8 ng/l		HPLC-MS/MS
	Groundwater	France (Reh et al., 2013)		9.38 ng/l	HPLC-MS/MS
	Effluent	Spain (López-Serna et al., 2013)		62 ng/l	HPLC-MS/MS
	Effluent	USA (Lara-Martín et al., 2014)	25-78 ng/l		HPLC-MS
	Effluent	Switzerland (Alder et al., 2010)		30 ng/l	LC-MS
	Effluent	Sweden (Bendz et al., 2005)	73 ng/l	235 ng/l	UPLC-MS/MS
	Effluent	Spain (Biel-Maeso et al., 2018)	104 ng/l	136 ng/l	LC–QqLIT- MS
	Surface water	Spain (Gros et al., 2008)		8 ng/l	HPLC-MS
	Influent	Switzerland (Alder et al., 2010)	36 ng/l	61 ng/l	LC–QTRAP–MS
	Effluent	Alcala de Henares, Spain (Rosal et al., 2010)	36 ng/l	57 ng/l	LC–QTRAP–MS
	Lake	Alcala de Henares, Spain (Rosal et al., 2010)	1.3 ng/l	52 ng/l	LC–MS/MS
		Africa (Nantaba et al., 2024)			

Table S4. Global levels of environmental contamination by commonly used pain relievers.

Pharmaceutical	Source	Location	Average conc.	Max conc	Detection Method
Acetaminophen (ACE)	Marine sediments	Auckland, New Zealand (Murdoch)	7660 ng/kg		-
(paracetamol)	Biosolids	Kaikoura, New Zealand (Murdoch)	76000 ng/kg		-
	Influent	Lis river, Portugal (Paíga et al., 2016)	159,225 ng/L	615,135 ng/L	UHPLC-MS/MS
	Effluent	Lis river, Portugal (Paíga et al., 2016)	1723 ng/L	2463 ng/L	UHPLC-MS/MS
	Surface water	Lagos, Nigeria (Folarin et al., 2019)	0.075 µg/l		HPLC-UV
	Surface water	Ologe, Nigeria (Folarin et al., 2019)	1.233 µg/l		HPLC-UV
	Influent	Tunisia (Moslah et al., 2018)	1208 ng/l		UPLC-MS/MS
	Effluent	Tunisia (Moslah et al., 2018)	409 ng/l		UPLC-MS/MS
	Downstream	South Africa (Archer et al., 2017)	63.7 ng/l		UPLC-MS
	Upstream	South Africa (Archer et al., 2017)	20.8 ng/l		UPLC-MS
	Influent	South Africa (Kanama et al., 2018)	4979 ng/l	119500 ng/l	LC-MS/MS
	Effluent	South Africa (Kanama et al., 2018)	750 ng/l	11390 ng/l	LC-MS/MS
	Effluent	Ebro river region, Spain (Gros et al., 2010)		293 ng/l	HPLC–QTRAP- MS
	River	Ebro river region, Spain (Gros et al., 2010)		143 ng/l	HPLC–QTRAP- MS
	Influent	Alcala de Henares, Spain (Rosal et al., 2010)	23202 ng/l	37458 ng/l	LC–QTRAP–MS
	Santo bay	Sao Paulo, Brazil (Pereira et al., 2016)	8.4 ng/l		LC–MS/MS
	Lake	Africa (Nantaba et al., 2024)	1.1 ng/l	1.7 ng/l	LC–MS/MS
Diclofenac (DFC)	Marine sediments	Auckland, New Zealand (Murdoch)	1.95 µg/kg		-

	Biosolids	Kaikoura, New Zealand (Murdoch)	8 µg/kg		-
	Influent	Lis river, Portugal (Paíga et al., 2016)	972 ng/L	972 ng/L	UHPLC-MS/MS
	Effluent	Lis river, Portugal (Paíga et al., 2016)	165 ng/L	144 ng/L	UHPLC-MS/MS
	Effluent	Spain (Loos et al., 2013)	50 ng/l	174 ng/l	SPE-LC-MS/MS
	Effluent	Lausanne, Switzerland (De la Cruz et al., 2012)	518 ng/l		UPLC-MS/MS
	Effluent		014 µg/l		MS
	Effluent	South Wales, UK (Kasprzyk-Hordern et al., 2009)	0.69 µg/l		LC-ESI-MS/MS
	Influent	Regensdorf, Switzerland (Singer et al., 2010)	1.45 µg/l		LC-ESI-MS/MS
	Effluent		0.33 µg/l	0.74 µg/l	UHPLC-MS/MS
	Effluent	Regensdorf, Switzerland (Singer et al., 2010)	0.9 µg/l		GS-MS
	Effluent	Castellon, Spain (Gracia-Lor et al., 2012)		1.9 µg/l	HPLC–QTRAP- MS
	River	South, Spain (Gómez et al., 2007)		120 ng/l	HPLC–QTRAP- MS
	Effluent	Ebro river region, Spain (Gros et al., 2010)	0.22 µg/l	0.43 µg/l	LC–QTRAP–MS
	Influent	Ebro river region, Spain (Gros et al., 2010)	232 ng/l	561 ng/l	LC–QTRAP–MS
	Surface water	Alcala de Henares, Spain (Rosal et al., 2010)	0.136 µg/l		HPLC-UV
	Surface water	Alcala de Henares, Spain (Rosal et al., 2010)	0.519 µg/l		HPLC-UV
	Santo bay		7.4 ng/l	19.4 ng/l	LC–MS/MS
	Seawater	Lagos, Nigeria (Folarin et al., 2019)	1-4 ng/l		-
	Effluent	Ologe, Nigeria (Folarin et al., 2019)	4411-34028 ng/l		-
	Seawater	Sao Paulo, Brazil (Pereira et al., 2016)	48 ng/l		-
	Effluent	Longyearbyen, Norway (Kallenborn et al., 2018)	25 ng/l		-

	Sea	Longyearbyen, Norway (Kallenborn et al., 2018)	0.25 ng/l	92.6 ng/l	LC–ESI–MS/MS
	Sea	Oslo, Norway (Kallenborn et al., 2018)	2 ng/l	9.2 ng/l	HPLC–MS/MS
	Sea	Oslo, Norway (Kallenborn et al., 2018)	0.006 ng/l	6.2 ng /l	GC-MS
	River	Poland (Borecka et al., 2015)	8 ng/l	195 ng/l	LC-MS
	Downstream	Germany (Nödler et al., 2014)	1461.5 ng/l		UPLC-MS
	Upstream	Germany (Weigel et al., 2002)	467.4 ng/l		UPLC-MS
	Influent	UK (Thomas and Hilton, 2004)	2380 ng/l	10340 ng/l	LC-MS/MS
	Effluent	South Africa (Archer et al., 2017)	270 ng/l	750 ng/l	LC-MS/MS
	Lake	South Africa (Archer et al., 2017)	1.3 ng/l	8.9 ng/l	LC–MS/MS
		South Africa (Kanama et al., 2018)			
		South Africa (Kanama et al., 2018)			
		Africa (Nantaba et al., 2024)			

Refrences

Al-Riyami, I.M., Ahmed, M., Al-Busaidi, A., Choudri, B., 2018. Antibiotics in wastewaters: a review with focus on Oman. Applied Water Science 8, 1-10.

Al Aukidy, M., Verlicchi, P., Jelic, A., Petrovic, M., Barcelò, D., 2012. Monitoring release of pharmaceutical compounds: occurrence and environmental risk assessment of two WWTP effluents and their receiving bodies in the Po Valley, Italy. Science of the Total Environment 438, 15-25.

Alder, A.C., Schaffner, C., Majewsky, M., Klasmeier, J., Fenner, K., 2010. Fate of β-blocker human pharmaceuticals in surface water: comparison of measured and simulated concentrations in the Glatt Valley Watershed, Switzerland. Water research 44, 936-948.

Anumol, T., Snyder, S.A., 2015. Rapid analysis of trace organic compounds in water by automated online solid-phase extraction coupled to liquid chromatography–tandem mass spectrometry. Talanta 132, 77-86.

Archer, E., Petrie, B., Kasprzyk-Hordern, B., Wolfaardt, G.M., 2017. The fate of pharmaceuticals and personal care products (PPCPs), endocrine disrupting contaminants (EDCs), metabolites and illicit drugs in a WWTW and environmental waters. Chemosphere 174, 437-446.

Arnnok, P., Singh, R.R., Burakham, R., Pérez-Fuentetaja, A., Aga, D.S., 2017. Selective uptake and bioaccumulation of antidepressants in fish from effluent-impacted Niagara River. Environmental science & technology 51, 10652-10662.

Ashfaq, M., Li, Y., Rehman, M.S.U., Zubair, M., Mustafa, G., Nazar, M.F., Yu, C.-P., Sun, Q., 2019. Occurrence, spatial variation and risk assessment of pharmaceuticals and personal care products in urban wastewater, canal surface water, and their sediments: A case study of Lahore, Pakistan. Science of the Total Environment 688, 653-663.

Aydin, E., Talinli, I., 2013. Analysis, occurrence and fate of commonly used pharmaceuticals and hormones in the Buyukcekmece Watershed, Turkey. Chemosphere 90, 2004-2012.

Azanu, D., Styrishave, B., Darko, G., Weisser, J.J., Abaidoo, R.C., 2018. Occurrence and risk assessment of antibiotics in water and lettuce in Ghana. Science of the Total Environment 622, 293-305.

Bataineh, H.N., Daradka, T., 2007. Effects of long-term use of fluoxetine on fertility parameters in adult male rats. Neuroendocrinology letters 28, 321-325.

Batt, A.L., Kim, S., Aga, D.S., 2007. Comparison of the occurrence of antibiotics in four full-scale wastewater treatment plants with varying designs and operations. Chemosphere 68, 428-435.

Bendz, D., Paxéus, N.A., Ginn, T.R., Loge, F.J., 2005. Occurrence and fate of pharmaceutically active compounds in the environment, a case study: Höje River in Sweden. Journal of hazardous materials 122, 195-204.

Biel-Maeso, M., Corada-Fernández, C., Lara-Martín, P.A., 2018. Monitoring the occurrence of pharmaceuticals in soils irrigated with reclaimed wastewater. Environmental Pollution 235, 312-321.

Bisognin, R.P., Wolff, D.B., Carissimi, E., Prestes, O.D., Zanella, R., 2021. Occurrence and fate of pharmaceuticals in effluent and sludge from a wastewater treatment plant in Brazil. Environmental Technology 42, 2292-2303.

Borecka, M., Siedlewicz, G., Haliński, Ł.P., Sikora, K., Pazdro, K., Stepnowski, P., Białk-Bielińska, A., 2015. Contamination of the southern Baltic Sea waters by the residues of selected pharmaceuticals: method development and field studies. Marine pollution bulletin 94, 62-71.

Borghi, A.A., Palma, M.S.A., 2014. Tetracycline: production, waste treatment and environmental impact assessment. Brazilian Journal of Pharmaceutical Sciences 50, 25-40.

Brunello, N., Blier, P., Judd, L.L., Mendlewicz, J., Nelson, C.J., Souery, D., Zohar, J., Racagni, G., 2003. Noradrenaline in mood and anxiety disorders: basic and clinical studies. International clinical psychopharmacology 18, 191-202.

Bueno, M.M., Gomez, M., Herrera, S., Hernando, M., Agüera, A., Fernández-Alba, A., 2012. Occurrence and persistence of organic emerging contaminants and priority pollutants in five sewage treatment plants of Spain: two years pilot survey monitoring. Environmental Pollution 164, 267-273.

Calamari, D., Zuccato, E., Castiglioni, S., Bagnati, R., Fanelli, R., 2003. Strategic survey of therapeutic drugs in the rivers Po and Lambro in northern Italy. Environmental science & technology 37, 1241-1248.
Carraro, E., Bonetta, S., Bertino, C., Lorenzi, E., Bonetta, S., Gilli, G., 2016. Hospital effluents management: chemical, physical, microbiological risks and legislation in different countries. Journal of Environmental Management 168, 185-199.
Castiglioni, S., Bagnati, R., Calamari, D., Fanelli, R., Zuccato, E., 2005. A multiresidue analytical method using solid-phase extraction and high-pressure liquid chromatography tandem mass spectrometry to measure pharmaceuticals of different therapeutic classes in urban wastewaters. Journal of Chromatography A 1092, 206-215.
Chiffre, A., Degiorgi, F., Buleté, A., Spinner, L., Badot, P.-M., 2016. Occurrence of pharmaceuticals in WWTP effluents and their impact in a karstic rural catchment of Eastern France. Environmental Science and Pollution Research 23, 25427-25441.
de Jesus Gaffney, V., Cardoso, V.V., Cardoso, E., Teixeira, A.P., Martins, J., Benoliel, M.J., Almeida, C.M.M., 2017. Occurrence and behaviour of pharmaceutical compounds in a Portuguese wastewater treatment plant: Removal efficiency through conventional treatment processes. Environmental Science and Pollution Research 24, 14717-14734.
De la Cruz, N., Giménez, J., Esplugas, S., Grandjean, D., De Alencastro, L., Pulgarin, C., 2012. Degradation of 32 emergent contaminants by UV and neutral photo-fenton in domestic wastewater effluent previously treated by activated sludge. Water research 46, 1947-1957.
Duarte, I.A., Reis-Santos, P., Novais, S.C., Rato, L.D., Lemos, M.F., Freitas, A., Pouca, A.S.V., Barbosa, J., Cabral, H.N., Fonseca, V.F., 2020. Depressed, hypertense and sore: Long-term effects of fluoxetine, propranolol and diclofenac exposure in a top predator fish. Science of the Total Environment 712, 136564.
Elizalde-Velázquez, A., Gómez-Oliván, L.M., Galar-Martínez, M., Islas-Flores, H., Dublán-García, O., SanJuan-Reyes, N., 2016. Amoxicillin in the aquatic environment, its fate and environmental risk. Environmental health risk-hazardous factors to living species 1, 247-267.
Estrada-Arriaga, E.B., Cortés-Muñoz, J.E., González-Herrera, A., Calderón-Mólgora, C.G., de Lourdes Rivera-Huerta, M., Ramírez-Camperos, E., Montellano-Palacios, L., Gelover-Santiago, S.L., Pérez-Castrejón, S., Cardoso-Vigueros, L., 2016. Assessment of full-scale biological nutrient removal systems upgraded with physico-chemical processes for the removal of emerging pollutants present in wastewaters from Mexico. Science of the Total Environment 571, 1172-1182.
Faleye, A., Adegoke, A., Ramluckan, K., Fick, J., Bux, F., Stenström, T., 2019. Concentration and reduction of antibiotic residues in selected wastewater treatment plants and receiving waterbodies in Durban, South Africa. Science of the Total Environment 678, 10-20.
Fick, J., Söderström, H., Lindberg, R.H., Phan, C., Tysklind, M., Larsson, D.J., 2009. Contamination of surface, ground, and drinking water from pharmaceutical production. Environmental toxicology and chemistry 28, 2522-2527.
Fitzsimmons, P.N., Fernandez, J.D., Hoffman, A.D., Butterworth, B.C., Nichols, J.W., 2001. Branchial elimination of superhydrophobic organic compounds by rainbow trout (Oncorhynchus mykiss). Aquatic toxicology 55, 23-34.
Folarin, O., Otitoloju, A., Amaeze, N., Saliu, J., 2019. Occurrence of acetaminophen, amoxicillin, diclofenac and methylparaben in Lagos and Ologe Lagoons, Lagos, Nigeria. Journal of Applied Sciences and Environmental Management 23, 2143-2149.
Fraher, D., Hodge, J., Collier, F., McMillan, J., Kennedy, R., Ellis, M., Nicholson, G.C., Walder, K., Dodd, S., Berk, M., 2016. Citalopram and sertraline exposure compromises embryonic bone development. Molecular psychiatry 21, 656-664.
Gómez, M.J., Bueno, M.M., Lacorte, S., Fernández-Alba, A.R., Agüera, A., 2007. Pilot survey monitoring pharmaceuticals and related compounds in a sewage treatment plant located on the Mediterranean coast. Chemosphere 66, 993-1002.

Gornik, T., Kovacic, A., Heath, E., Hollender, J., Kosjek, T., 2020. Biotransformation study of antidepressant sertraline and its removal during biological wastewater treatment. Water research 181, 115864.
Gracia-Lor, E., Sancho, J.V., Serrano, R., Hernández, F., 2012. Occurrence and removal of pharmaceuticals in wastewater treatment plants at the Spanish Mediterranean area of Valencia. Chemosphere 87, 453-462.
Gros, M., Petrović, M., Ginebreda, A., Barceló, D., 2010. Removal of pharmaceuticals during wastewater treatment and environmental risk assessment using hazard indexes. Environment international 36, 15-26.
Gros, M., Pizzolato, T.-M., Petrović, M., de Alda, M.J.L., Barceló, D., 2008. Trace level determination of β-blockers in waste waters by highly selective molecularly imprinted polymers extraction followed by liquid chromatography–quadrupole-linear ion trap mass spectrometry. Journal of Chromatography A 1189, 374-384.
Hanna, N., Sun, P., Sun, Q., Li, X., Yang, X., Ji, X., Zou, H., Ottoson, J., Nilsson, L.E., Berglund, B., 2018. Presence of antibiotic residues in various environmental compartments of Shandong province in eastern China: its potential for resistance development and ecological and human risk. Environment international 114, 131-142.
Harnisz, M., Korzeniewska, E., Gołaś, I., 2015. The impact of a freshwater fish farm on the community of tetracycline-resistant bacteria and the structure of tetracycline resistance genes in river water. Chemosphere 128, 134-141.
Holzschuh, J., Ryu, S., Aberger, F., Driever, W., 2001. Dopamine transporter expression distinguishes dopaminergic neurons from other catecholaminergic neurons in the developing zebrafish embryo. Mechanisms of development 101, 237-243.
Hou, J., Wang, C., Mao, D., Luo, Y., 2016. The occurrence and fate of tetracyclines in two pharmaceutical wastewater treatment plants of Northern China. Environmental Science and Pollution Research 23, 1722-1731.
Huggett, D., Cook, J., Ericson, J., Williams, R., 2003. A theoretical model for utilizing mammalian pharmacology and safety data to prioritize potential impacts of human pharmaceuticals to fish. Human and Ecological Risk Assessment 9, 1789-1799.
Jia, J., Guan, Y., Cheng, M., Chen, H., He, J., Wang, S., Wang, Z., 2018. Occurrence and distribution of antibiotics and antibiotic resistance genes in Ba River, China. Science of the Total Environment 642, 1136-1144.
Kafaei, R., Papari, F., Seyedabadi, M., Sahebi, S., Tahmasebi, R., Ahmadi, M., Sorial, G.A., Asgari, G., Ramavandi, B., 2018. Occurrence, distribution, and potential sources of antibiotics pollution in the water-sediment of the northern coastline of the Persian Gulf, Iran. Science of the Total Environment 627, 703-712.
Kairigo, P., Ngumba, E., Sundberg, L.-R., Gachanja, A., Tuhkanen, T., 2020. Occurrence of antibiotics and risk of antibiotic resistance evolution in selected Kenyan wastewaters, surface waters and sediments. Science of the Total Environment 720, 137580.
Kallenborn, R., Brorström-Lundén, E., Reiersen, L.-O., Wilson, S., 2018. Pharmaceuticals and personal care products (PPCPs) in Arctic environments: indicator contaminants for assessing local and remote anthropogenic sources in a pristine ecosystem in change. Environmental Science and Pollution Research 25, 33001-33013.
Kanama, K.M., Daso, A.P., Mpenyana-Monyatsi, L., Coetzee, M.A., 2018. Assessment of pharmaceuticals, personal care products, and hormones in wastewater treatment plants receiving inflows from health facilities in North West Province, South Africa. Journal of toxicology 2018, 3751930.
Kasprzyk-Hordern, B., Dinsdale, R.M., Guwy, A.J., 2007. Multi-residue method for the determination of basic/neutral pharmaceuticals and illicit drugs in surface water by solid-phase extraction and ultra performance liquid chromatography–positive electrospray ionisation tandem mass spectrometry. Journal of Chromatography A 1161, 132-145.

Kasprzyk-Hordern, B., Dinsdale, R.M., Guwy, A.J., 2009. The removal of pharmaceuticals, personal care products, endocrine disruptors and illicit drugs during wastewater treatment and its impact on the quality of receiving waters. Water research 43, 363-380.
Khan, M.H., Bae, H., Jung, J.-Y., 2010. Tetracycline degradation by ozonation in the aqueous phase: proposed degradation intermediates and pathway. Journal of hazardous materials 181, 659-665.
Klančar, A., Trontelj, J., Roškar, R., 2018. Development of a multi-residue method for monitoring 44 pharmaceuticals in slovene surface water by SPE-LC-MS/MS. Water, Air, & Soil Pollution 229, 1-18.
Koba, O., Grabicova, K., Cerveny, D., Turek, J., Kolarova, J., Randak, T., Zlabek, V., Grabic, R., 2018. Transport of pharmaceuticals and their metabolites between water and sediments as a further potential exposure for aquatic organisms. Journal of hazardous materials 342, 401-407.
Kosma, C.I., Kapsi, M.G., Konstas, P.-S.G., Trantopoulos, E.P., Boti, V.I., Konstantinou, I.K., Albanis, T.A., 2020. Assessment of multiclass pharmaceutical active compounds (PhACs) in hospital WWTP influent and effluent samples by UHPLC-Orbitrap MS: Temporal variation, removals and environmental risk assessment. Environmental Research 191, 110152.
Kumar, R., Sarmah, A.K., Padhye, L.P., 2019. Fate of pharmaceuticals and personal care products in a wastewater treatment plant with parallel secondary wastewater treatment train. Journal of Environmental Management 233, 649-659.
Lara-Martín, P.A., González-Mazo, E., Petrovic, M., Barceló, D., Brownawell, B.J., 2014. Occurrence, distribution and partitioning of nonionic surfactants and pharmaceuticals in the urbanized Long Island Sound Estuary (NY). Marine pollution bulletin 85, 710-719.
Larsson, D.J., de Pedro, C., Paxeus, N., 2007. Effluent from drug manufactures contains extremely high levels of pharmaceuticals. Journal of hazardous materials 148, 751-755.
Lee, H.-J., Kim, K.Y., Hamm, S.-Y., Kim, M., Kim, H.K., Oh, J.-E., 2019. Occurrence and distribution of pharmaceutical and personal care products, artificial sweeteners, and pesticides in groundwater from an agricultural area in Korea. Science of the Total Environment 659, 168-176.
Loos, R., Carvalho, R., António, D.C., Comero, S., Locoro, G., Tavazzi, S., Paracchini, B., Ghiani, M., Lettieri, T., Blaha, L., 2013. EU-wide monitoring survey on emerging polar organic contaminants in wastewater treatment plant effluents. Water research 47, 6475-6487.
López-Serna, R., Jurado, A., Vázquez-Suñé, E., Carrera, J., Petrović, M., Barceló, D., 2013. Occurrence of 95 pharmaceuticals and transformation products in urban groundwaters underlying the metropolis of Barcelona, Spain. Environmental Pollution 174, 305-315.
Lu, S., Lin, C., Lei, K., Wang, B., Xin, M., Gu, X., Cao, Y., Liu, X., Ouyang, W., He, M., 2020. Occurrence, spatiotemporal variation, and ecological risk of antibiotics in the water of the semi-enclosed urbanized Jiaozhou Bay in eastern China. Water research 184, 116187.
Lundborg, C.S., Tamhankar, A.J., 2017. Antibiotic residues in the environment of South East Asia. Bmj 358.
Ma, L.-d., Li, J., Li, J.-j., Liu, M., Yan, D.-z., Shi, W.-y., Xu, G., 2018. Occurrence and source analysis of selected antidepressants and their metabolites in municipal wastewater and receiving surface water. Environmental Science: Processes & Impacts 20, 1020-1029.
Mackie, R.I., Koike, S., Krapac, I., Chee-Sanford, J., Maxwell, S., Aminov, R.I., 2006. Tetracycline residues and tetracycline resistance genes in groundwater impacted by swine production facilities. Animal biotechnology 17, 157-176.
Marrone, R.L., Pray, S.L., Bridges, C.C., 1966. Norepinephrine elicitation of aggressive display responses in Betta splendens. Psychonomic Science 5, 207-208.
Mashile, P.P., Nomngongo, P.N., 2021. Magnetic cellulose-chitosan nanocomposite for simultaneous removal of emerging contaminants: Adsorption kinetics and equilibrium studies. Gels 7, 190.
Melvin, S.D., 2017. Effect of antidepressants on circadian rhythms in fish: Insights and implications regarding the design of behavioural toxicity tests. Aquatic toxicology 182, 20-30.
Metcalfe, C.D., Chu, S., Judt, C., Li, H., Oakes, K.D., Servos, M.R., Andrews, D.M., 2010. Antidepressants and their metabolites in municipal wastewater, and downstream exposure in an urban watershed. Environmental toxicology and chemistry 29, 79-89.

Miège, C., Favier, M., Brosse, C., Canler, J.-P., Coquery, M., 2006. Occurrence of betablockers in effluents of wastewater treatment plants from the Lyon area (France) and risk assessment for the downstream rivers. Talanta 70, 739-744.
Mole, R.A., Brooks, B.W., 2019. Global scanning of selective serotonin reuptake inhibitors: occurrence, wastewater treatment and hazards in aquatic systems. Environmental Pollution 250, 1019-1031.
Moslah, B., Hapeshi, E., Jrad, A., Fatta-Kassinos, D., Hedhili, A., 2018. Pharmaceuticals and illicit drugs in wastewater samples in north-eastern Tunisia. Environmental Science and Pollution Research 25, 18226-18241.
Mostafa, A., Shaaban, H., Alqarni, A., Al-Ansari, R., Alrashidi, A., Al-Sultan, F., Alsulaiman, M., Alsaif, F., Aga, O., 2023. Multi-class determination of pharmaceuticals as emerging contaminants in wastewater from Eastern Province, Saudi Arabia using eco-friendly SPE-UHPLC-MS/MS: Occurrence, removal and environmental risk assessment. Microchemical Journal 187, 108453.
Murata, A., Takada, H., Mutoh, K., Hosoda, H., Harada, A., Nakada, N., 2011. Nationwide monitoring of selected antibiotics: distribution and sources of sulfonamides, trimethoprim, and macrolides in Japanese rivers. Science of the Total Environment 409, 5305-5312.
Murdoch, K., Pharmaceutical Pollution in the Environment: Issues for Australia, New Zealand and Pacific Island countries, 2015.
Naderi, M., Jamwal, A., Chivers, D.P., Niyogi, S., 2016. Modulatory effects of dopamine receptors on associative learning performance in zebrafish (Danio rerio). Behavioural brain research 303, 109-119.
Nakamura, Y., Yamamoto, H., Sekizawa, J., Kondo, T., Hirai, N., Tatarazako, N., 2008. The effects of pH on fluoxetine in Japanese medaka (Oryzias latipes): acute toxicity in fish larvae and bioaccumulation in juvenile fish. Chemosphere 70, 865-873.
Nantaba, F., Wasswa, J., Kylin, H., Bouwman, H., Palm, W.-U., Kümmerer, K., 2024. Spatial trends and ecotoxic risk assessment of selected pharmaceuticals in sediments from Lake Victoria, Uganda, East Africa. Science of the Total Environment 906, 167348.
Nantaba, F., Wasswa, J., Kylin, H., Palm, W.-U., Bouwman, H., Kümmerer, K., 2020. Occurrence, distribution, and ecotoxicological risk assessment of selected pharmaceutical compounds in water from Lake Victoria, Uganda. Chemosphere 239, 124642.
Nödler, K., Voutsa, D., Licha, T., 2014. Polar organic micropollutants in the coastal environment of different marine systems. Marine pollution bulletin 85, 50-59.
Ohoro, C.R., Adeniji, A.O., Semerjian, L., Okoh, O.O., Okoh, A.I., 2021. Occurrence and distribution of pharmaceuticals in surface water and sediment of Buffalo and Sundays River estuaries, South Africa and their ecological risk assessment. Emerging Contaminants 7, 187-195.
Oosterhuis, M., Sacher, F., Ter Laak, T.L., 2013. Prediction of concentration levels of metformin and other high consumption pharmaceuticals in wastewater and regional surface water based on sales data. Science of the Total Environment 442, 380-388.
Paíga, P., Santos, L.H., Ramos, S., Jorge, S., Silva, J.G., Delerue-Matos, C., 2016. Presence of pharmaceuticals in the Lis river (Portugal): Sources, fate and seasonal variation. Science of the Total Environment 573, 164-177.
Papageorgiou, M., Kosma, C., Lambropoulou, D., 2016. Seasonal occurrence, removal, mass loading and environmental risk assessment of 55 pharmaceuticals and personal care products in a municipal wastewater treatment plant in Central Greece. Science of the Total Environment 543, 547-569.
Patel, M., Kumar, R., Kishor, K., Mlsna, T., Pittman Jr, C.U., Mohan, D., 2019. Pharmaceuticals of emerging concern in aquatic systems: chemistry, occurrence, effects, and removal methods. Chemical reviews 119, 3510-3673.
Pereira, C.D.S., Maranho, L.A., Cortez, F.S., Pusceddu, F.H., Santos, A.R., Ribeiro, D.A., Cesar, A., Guimarães, L.L., 2016. Occurrence of pharmaceuticals and cocaine in a Brazilian coastal zone. Science of the Total Environment 548, 148-154.
Pivetta, R.C., Rodrigues-Silva, C., Ribeiro, A.R., Rath, S., 2020. Tracking the occurrence of psychotropic pharmaceuticals in Brazilian wastewater treatment plants and surface water, with assessment of environmental risks. Science of the Total Environment 727, 138661.

Reh, R., Licha, T., Geyer, T., Nödler, K., Sauter, M., 2013. Occurrence and spatial distribution of organic micro-pollutants in a complex hydrogeological karst system during low flow and high flow periods, results of a two-year study. Science of the Total Environment 443, 438-445.
Rivera-Jaimes, J.A., Postigo, C., Melgoza-Alemán, R.M., Aceña, J., Barceló, D., de Alda, M.L., 2018. Study of pharmaceuticals in surface and wastewater from Cuernavaca, Morelos, Mexico: occurrence and environmental risk assessment. Science of the Total Environment 613, 1263-1274.
Roberts, J., Kumar, A., Du, J., Hepplewhite, C., Ellis, D.J., Christy, A.G., Beavis, S.G., 2016. Pharmaceuticals and personal care products (PPCPs) in Australia's largest inland sewage treatment plant, and its contribution to a major Australian river during high and low flow. Science of the Total Environment 541, 1625-1637.
Roberts, P.H., Thomas, K.V., 2006. The occurrence of selected pharmaceuticals in wastewater effluent and surface waters of the lower Tyne catchment. Science of the Total Environment 356, 143-153.
Rosal, R., Rodríguez, A., Perdigón-Melón, J.A., Petre, A., García-Calvo, E., Gómez, M.J., Agüera, A., Fernández-Alba, A.R., 2010. Occurrence of emerging pollutants in urban wastewater and their removal through biological treatment followed by ozonation. Water research 44, 578-588.
Rúa-Gómez, P.C., Püttmann, W., 2012. Impact of wastewater treatment plant discharge of lidocaine, tramadol, venlafaxine and their metabolites on the quality of surface waters and groundwater. Journal of Environmental Monitoring 14, 1391-1399.
Schlüsener, M.P., Hardenbicker, P., Nilson, E., Schulz, M., Viergutz, C., Ternes, T.A., 2015. Occurrence of venlafaxine, other antidepressants and selected metabolites in the Rhine catchment in the face of climate change. Environmental Pollution 196, 247-256.
Schultz, W., 2007. Behavioral dopamine signals. Trends in neurosciences 30, 203-210.
Silva, L.J., Lino, C.M., Meisel, L.M., Pena, A., 2012. Selective serotonin re-uptake inhibitors (SSRIs) in the aquatic environment: an ecopharmacovigilance approach. Science of the Total Environment 437, 185-195.
Singer, H., Jaus, S., Hanke, I., Lück, A., Hollender, J., Alder, A.C., 2010. Determination of biocides and pesticides by on-line solid phase extraction coupled with mass spectrometry and their behaviour in wastewater and surface water. Environmental Pollution 158, 3054-3064.
Stewart, M., 2013. Pharmaceutical residues in the Auckland estuarine environment. Auckland Council.
Subedi, B., Kannan, K., 2015. Occurrence and fate of select psychoactive pharmaceuticals and antihypertensives in two wastewater treatment plants in New York State, USA. Science of the Total Environment 514, 273-280.
Thomas, K.V., Hilton, M.J., 2004. The occurrence of selected human pharmaceutical compounds in UK estuaries. Marine pollution bulletin 49, 436-444.
Thörnqvist, P.-O., McCarrick, S., Ericsson, M., Roman, E., Winberg, S., 2019. Bold zebrafish (Danio rerio) express higher levels of delta opioid and dopamine D2 receptors in the brain compared to shy fish. Behavioural brain research 359, 927-934.
Tran, N.H., Hoang, L., Nghiem, L.D., Nguyen, N.M.H., Ngo, H.H., Guo, W., Trinh, Q.T., Mai, N.H., Chen, H., Nguyen, D.D., 2019. Occurrence and risk assessment of multiple classes of antibiotics in urban canals and lakes in Hanoi, Vietnam. Science of the Total Environment 692, 157-174.
Valcárcel, Y., Alonso, S.G., Rodríguez-Gil, J., Maroto, R.R., Gil, A., Catalá, M., 2011. Analysis of the presence of cardiovascular and analgesic/anti-inflammatory/antipyretic pharmaceuticals in river-and drinking-water of the Madrid Region in Spain. Chemosphere 82, 1062-1071.
Vieno, N., Tuhkanen, T., Kronberg, L., 2007. Elimination of pharmaceuticals in sewage treatment plants in Finland. Water research 41, 1001-1012.
Vieno, N.M., Tuhkanen, T., Kronberg, L., 2006. Analysis of neutral and basic pharmaceuticals in sewage treatment plants and in recipient rivers using solid phase extraction and liquid chromatography–tandem mass spectrometry detection. Journal of Chromatography A 1134, 101-111.
Voloshenko-Rossin, A., Gasser, G., Cohen, K., Gun, J., Cumbal-Flores, L., Parra-Morales, W., Sarabia, F., Ojeda, F., Lev, O., 2015. Emerging pollutants in the Esmeraldas watershed in Ecuador: discharge and

attenuation of emerging organic pollutants along the San Pedro–Guayllabamba–Esmeraldas rivers. Environmental Science: Processes & Impacts 17, 41-53.
Vulliet, E., Cren-Olivé, C., 2011. Screening of pharmaceuticals and hormones at the regional scale, in surface and groundwaters intended to human consumption. Environmental Pollution 159, 2929-2934.
Wang, Z., Chen, Q., Zhang, J., Dong, J., Yan, H., Chen, C., Feng, R., 2019. Characterization and source identification of tetracycline antibiotics in the drinking water sources of the lower Yangtze River. Journal of Environmental Management 244, 13-22.
Watkinson, A., Murby, E., Costanzo, S., 2007. Removal of antibiotics in conventional and advanced wastewater treatment: implications for environmental discharge and wastewater recycling. Water research 41, 4164-4176.
Watkinson, A., Murby, E., Kolpin, D.W., Costanzo, S., 2009. The occurrence of antibiotics in an urban watershed: from wastewater to drinking water. Science of the Total Environment 407, 2711-2723.
Weigel, S., Kuhlmann, J., Hühnerfuss, H., 2002. Drugs and personal care products as ubiquitous pollutants: occurrence and distribution of clofibric acid, caffeine and DEET in the North Sea. Science of the Total Environment 295, 131-141.
Yuan, S.-F., Liu, Z.-H., Huang, R.-P., Yin, H., Dang, Z., 2016. Levels of six antibiotics used in China estimated by means of wastewater-based epidemiology. Water Science and Technology 73, 769-775.
Zhang, M., Liu, Y.-S., Zhao, J.-L., Liu, W.-R., He, L.-Y., Zhang, J.-N., Chen, J., He, L.-K., Zhang, Q.-Q., Ying, G.-G., 2018. Occurrence, fate and mass loadings of antibiotics in two swine wastewater treatment systems. Science of the Total Environment 639, 1421-1431.
